# Supplementary material for: Synthesis, Docking, and Machine Learning Studies of Some Novel Quinolinesulfonamides–Triazole Hybrids with Anticancer Activity
Source: Molecules. 2024 Jul 2;29(13):3158. doi: 10.3390/molecules29133158 (PMC11243625; doi:10.3390/molecules29133158)
Supplement: Supplementary file 1 [file molecules-29-03158-s001.zip › molecules-3072154-supplementary.pdf]

Synthesis, docking, and machine learning studies of some novel quinolinesulfonamides-triazole hybrids with anticancer activity

Krzysztof Marciniec, Justyna Nowakowska, Elwira Chrobak, Ewa Bębenek, Małgorzata Latocha

Spectroscopic data of compounds **4-14**.

1. ^{1}H NMR spectra

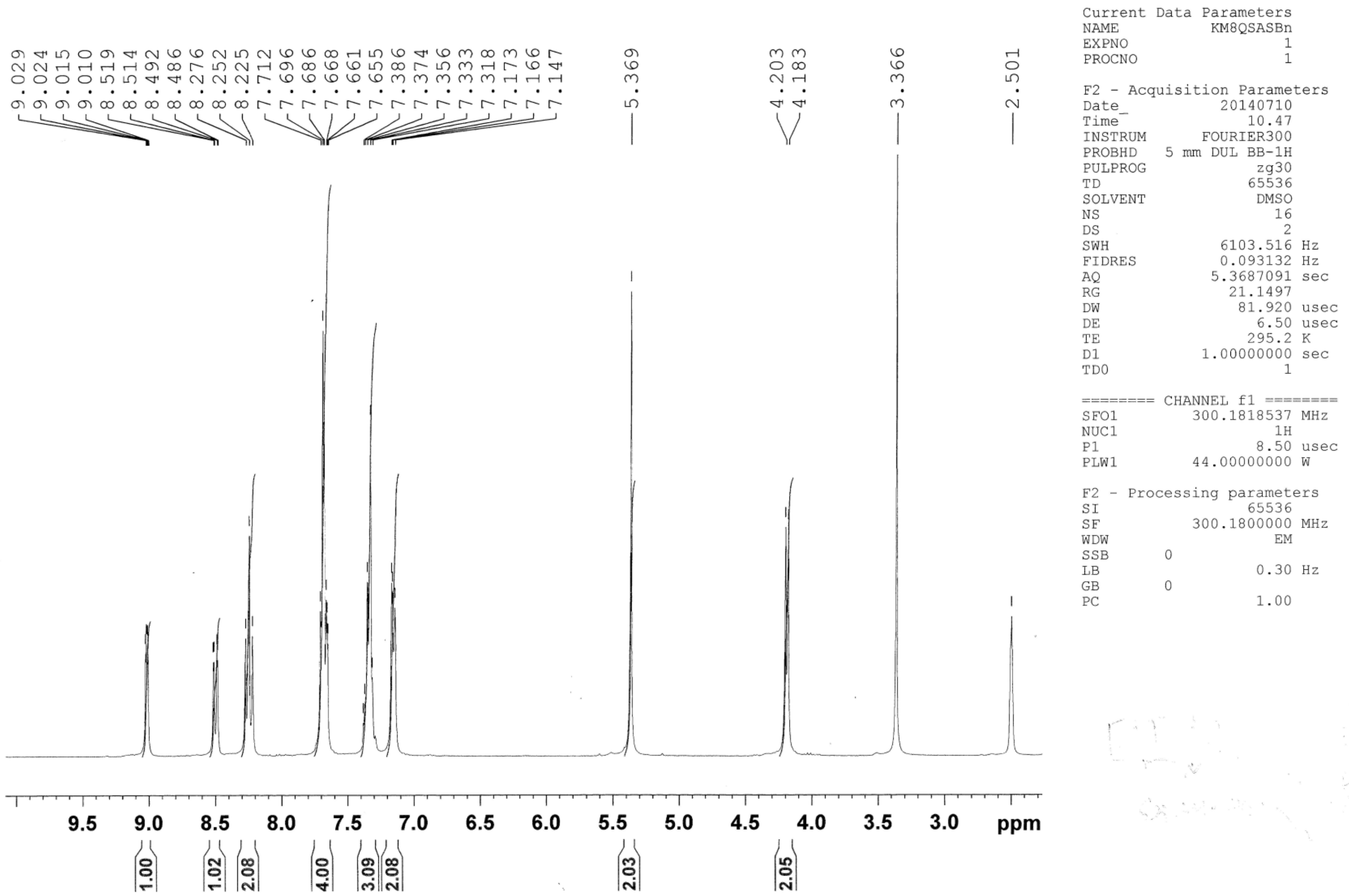

Figure 1. ^{1}H NMR spectra of compound **4a**

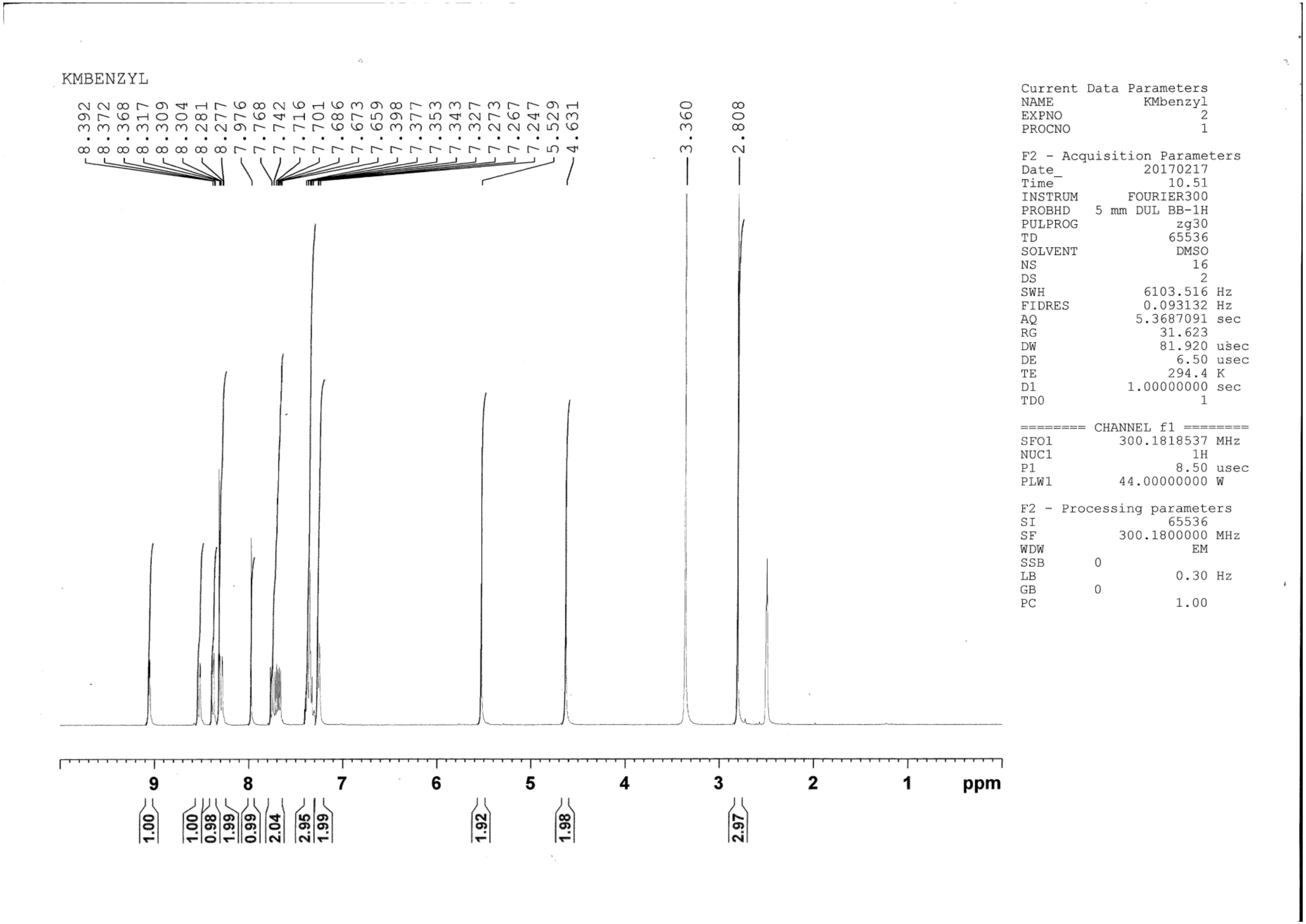

Figure 2. ^{1}H NMR spectra of compound **4b**

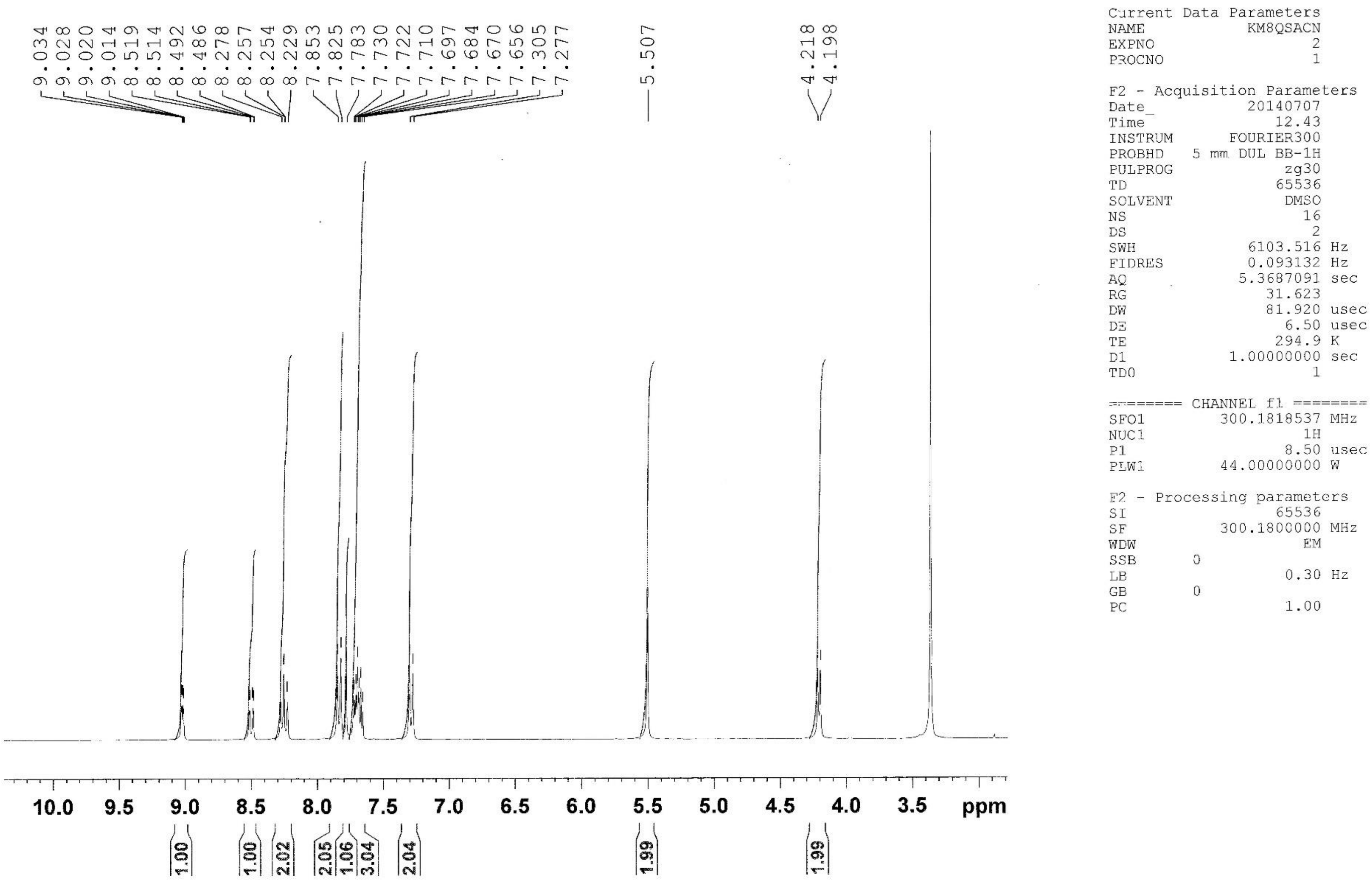

Figure 3. ^{1}H NMR spectra of compound **5a**

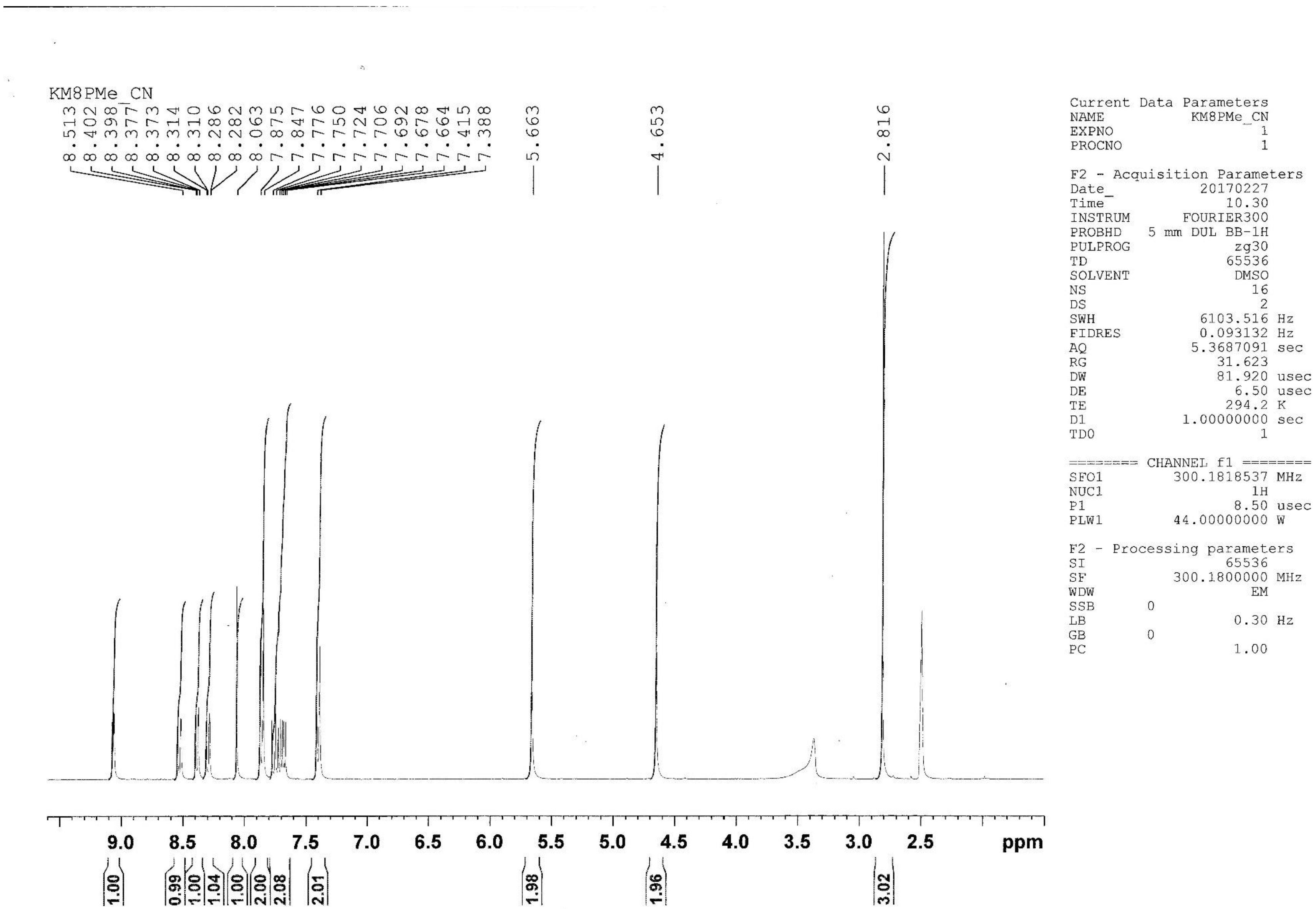

Figure 4. ^{1}H NMR spectra of compound **5b**

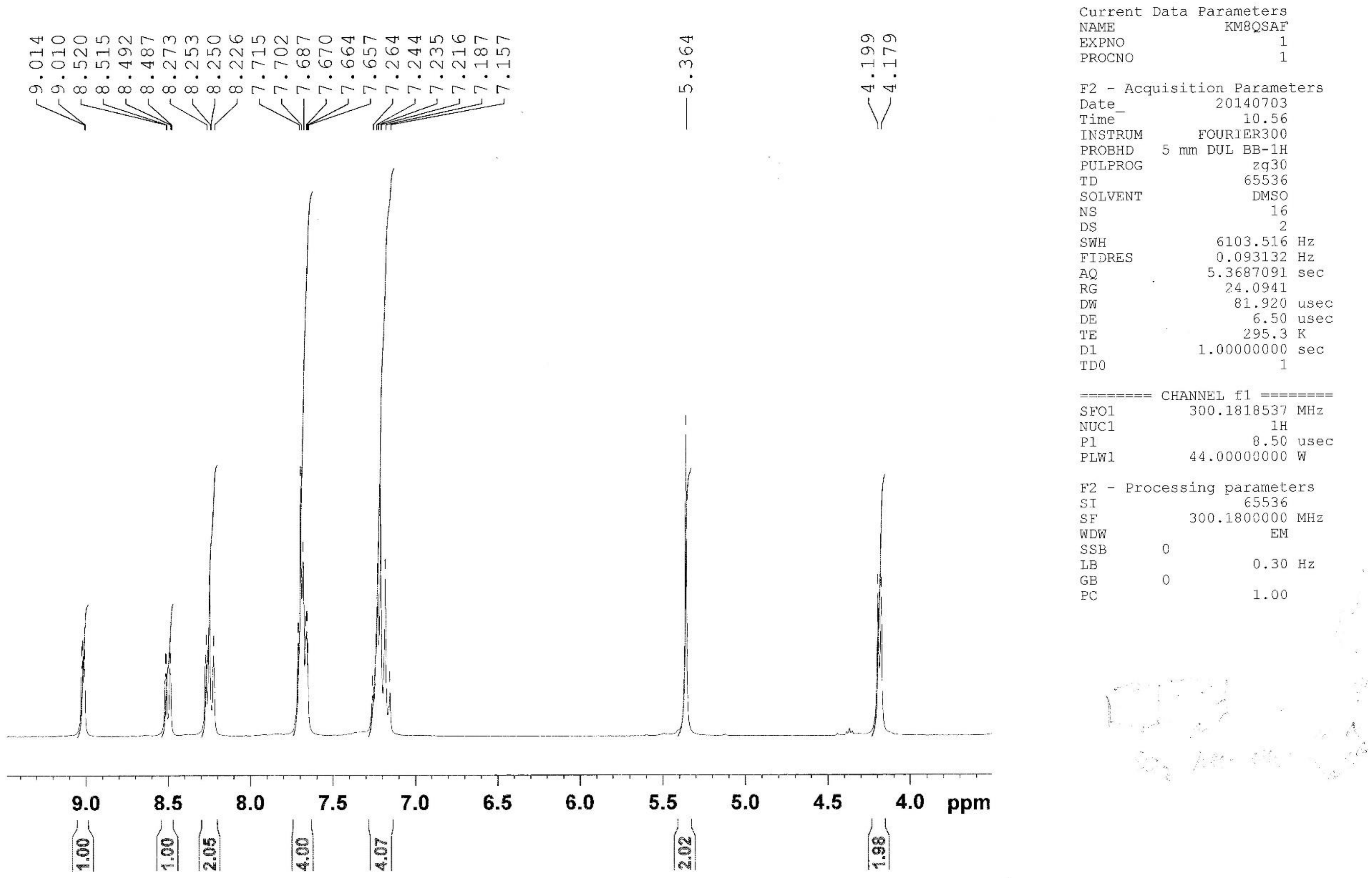

Figure 5. ^{1}H NMR spectra of compound **6a**

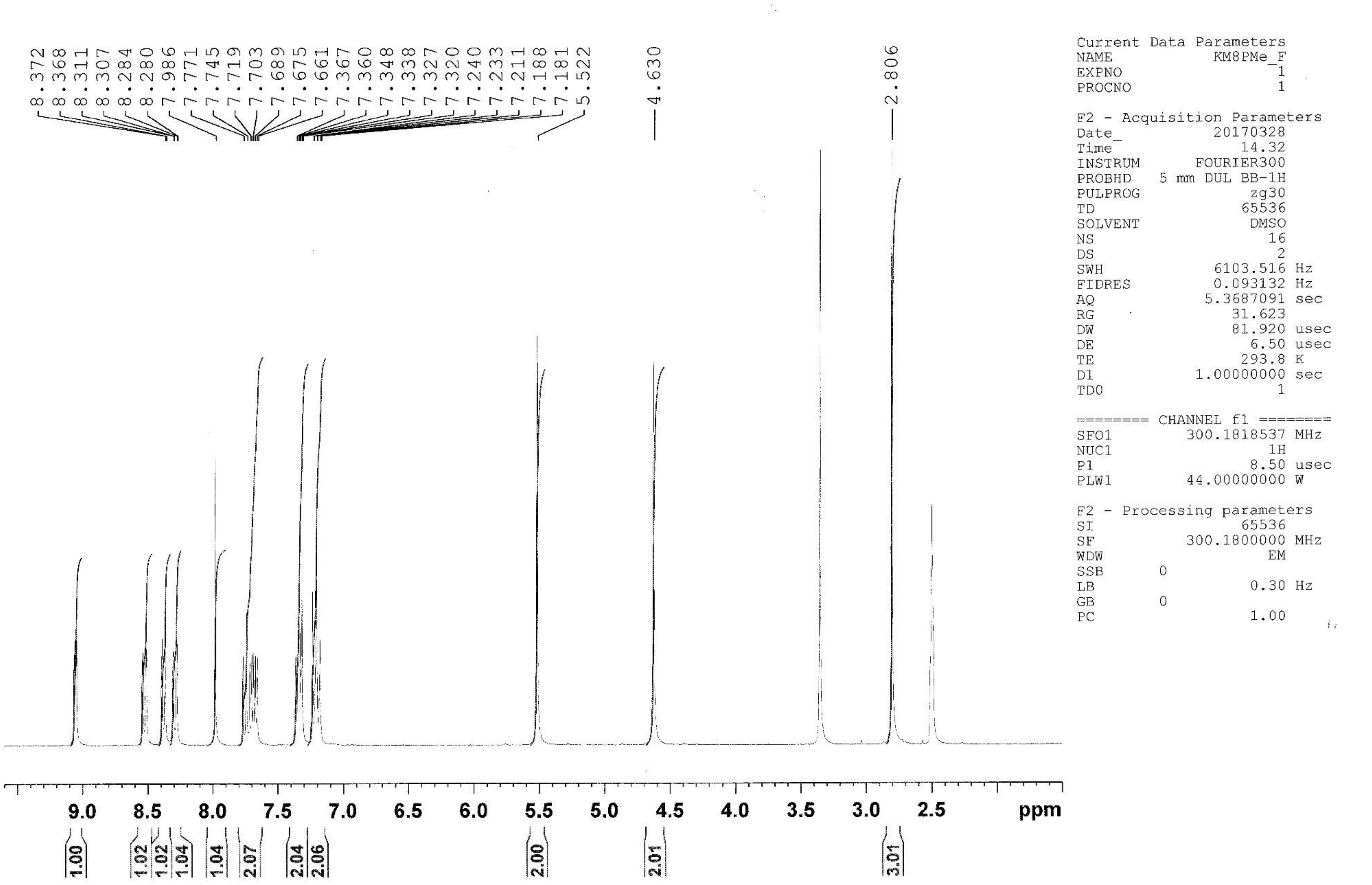

Figure 6. ^{1}H NMR spectra of compound **6b**

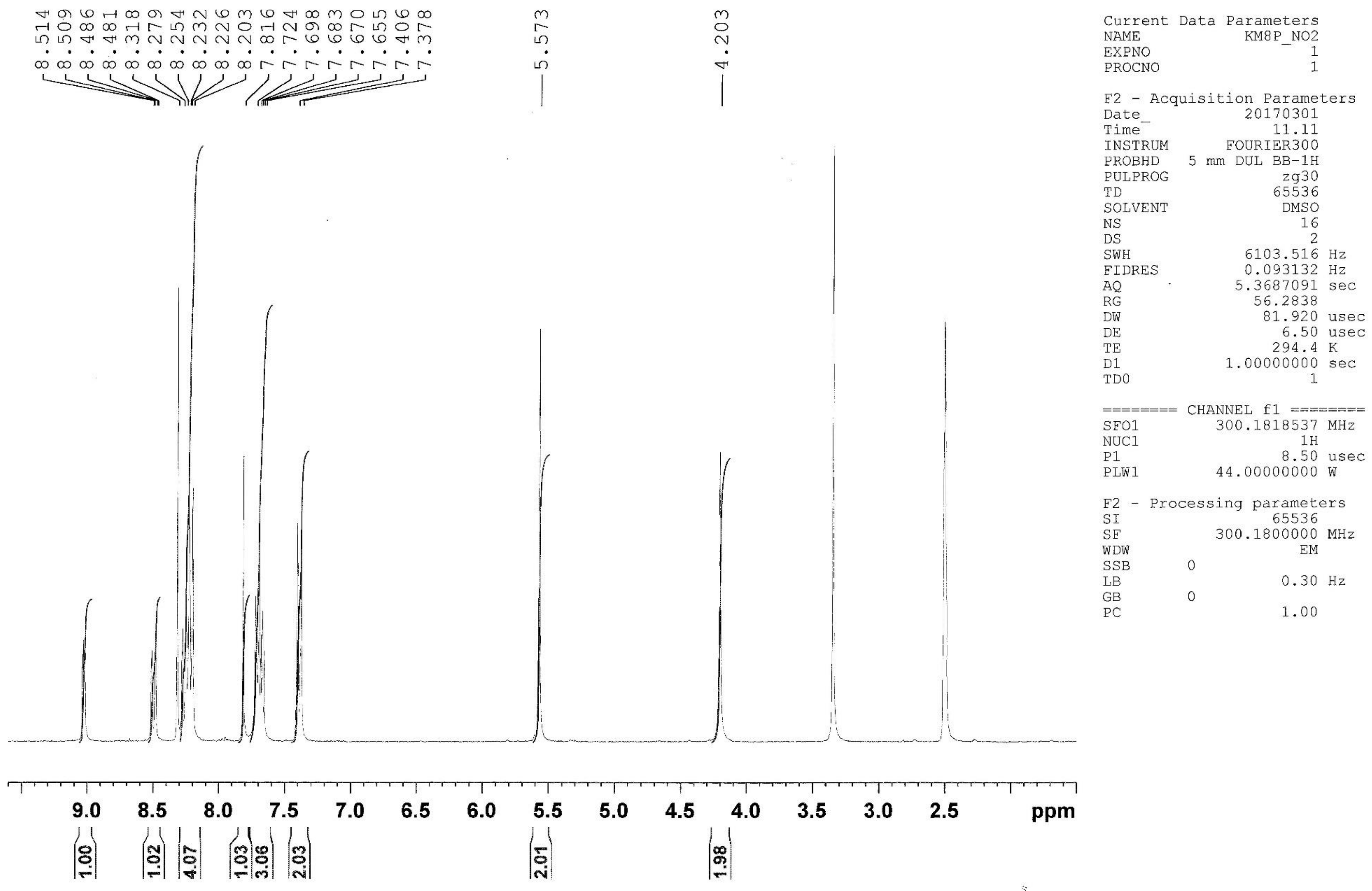

Figure 7. ^{1}H NMR spectra of compound **7a**

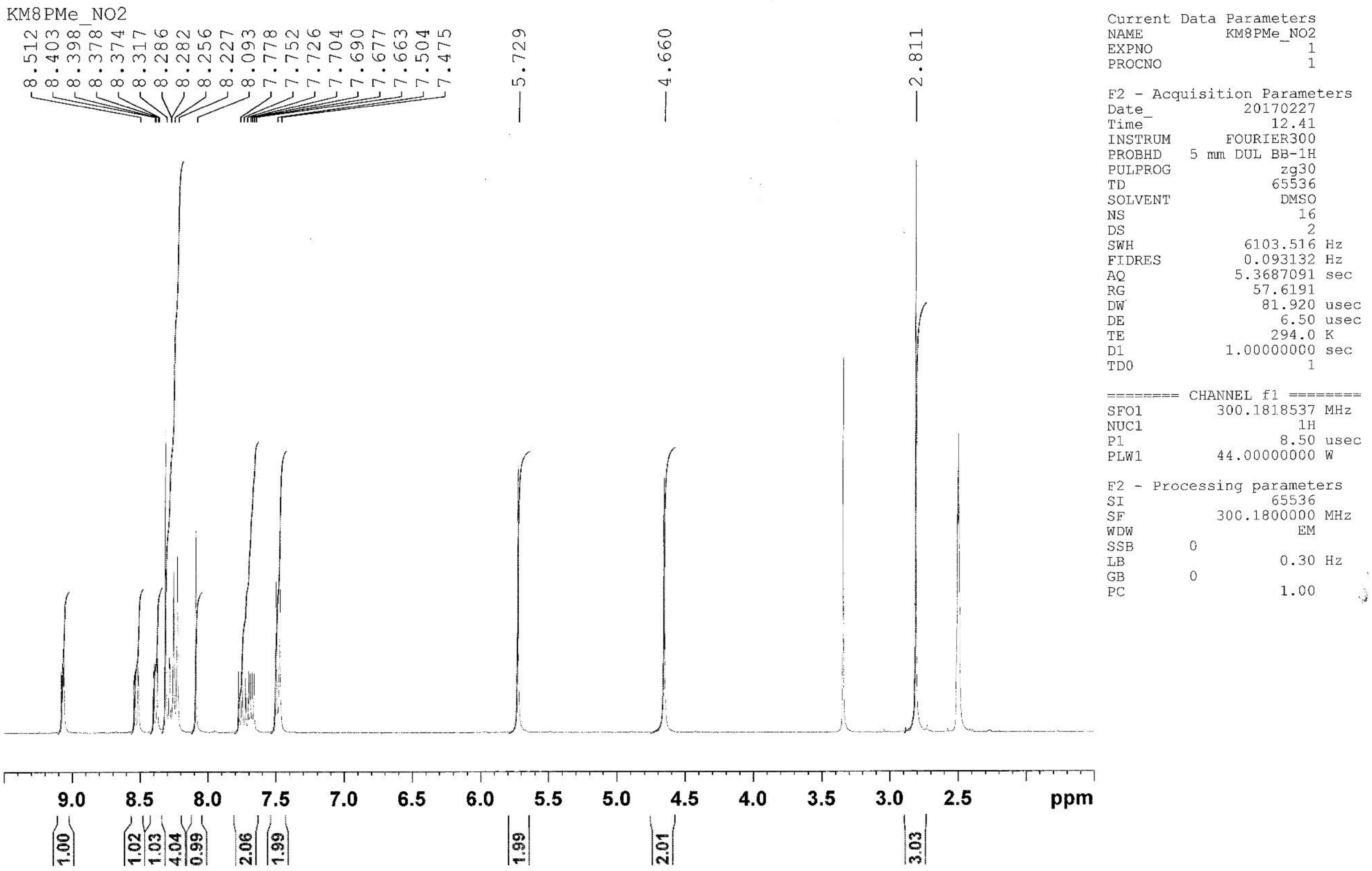

Figure 8. ^{1}H NMR spectra of compound **7b**

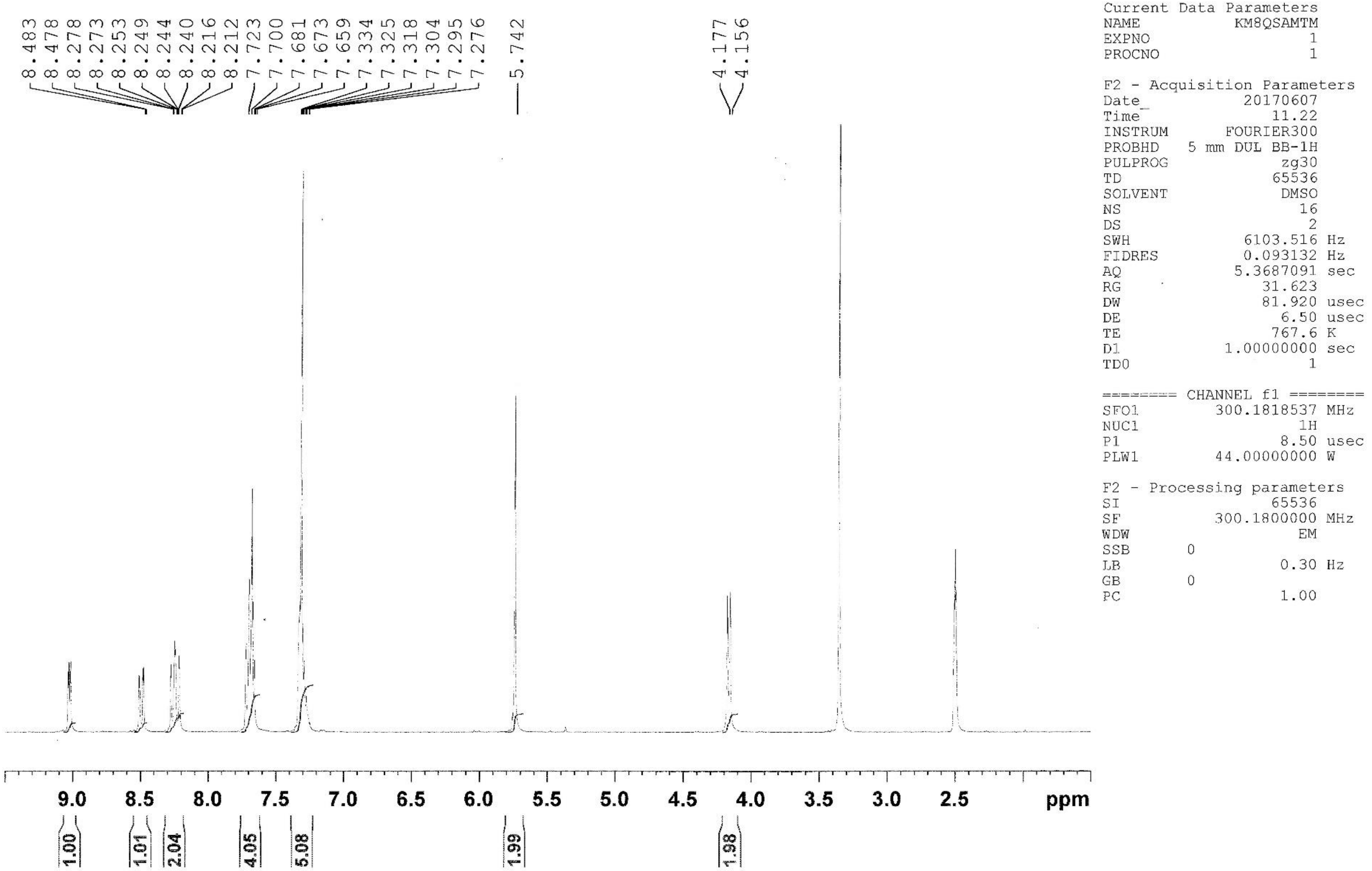

Figure 9. ^{1}H NMR spectra of compound **8a**

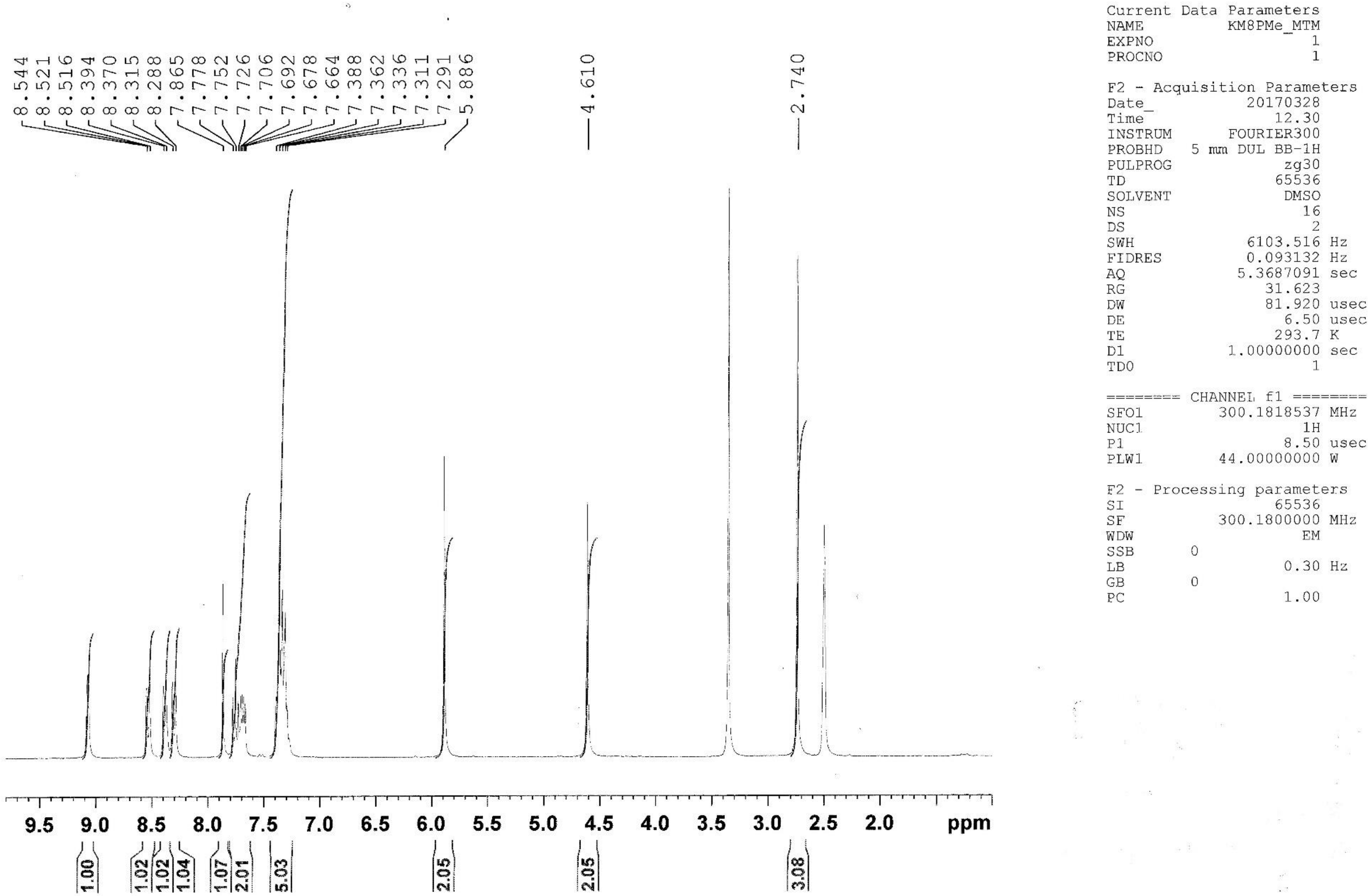

Figure 10. ^{1}H NMR spectra of compound **8b**

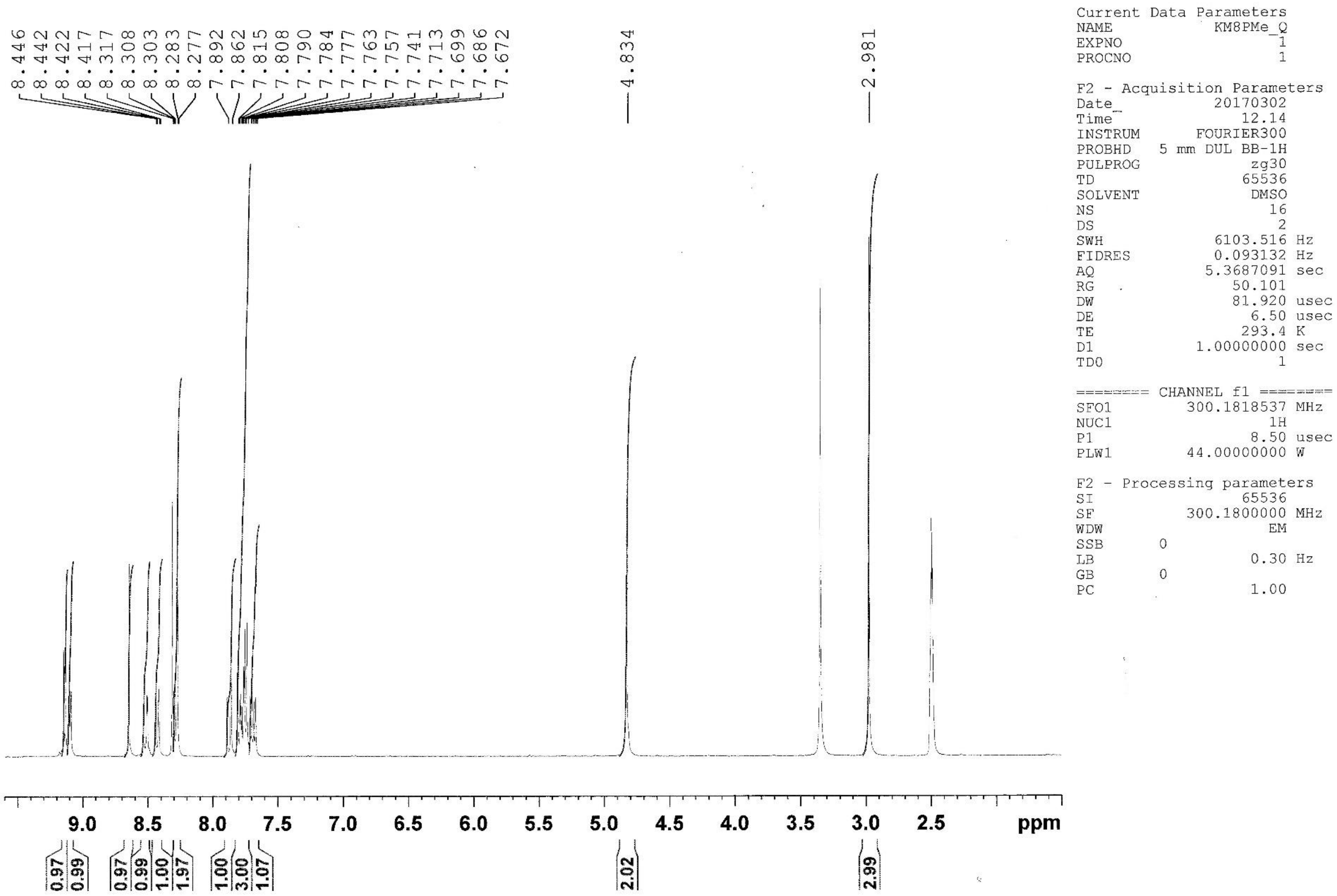

Figure 11. ^{1}H NMR spectra of compound **9b**

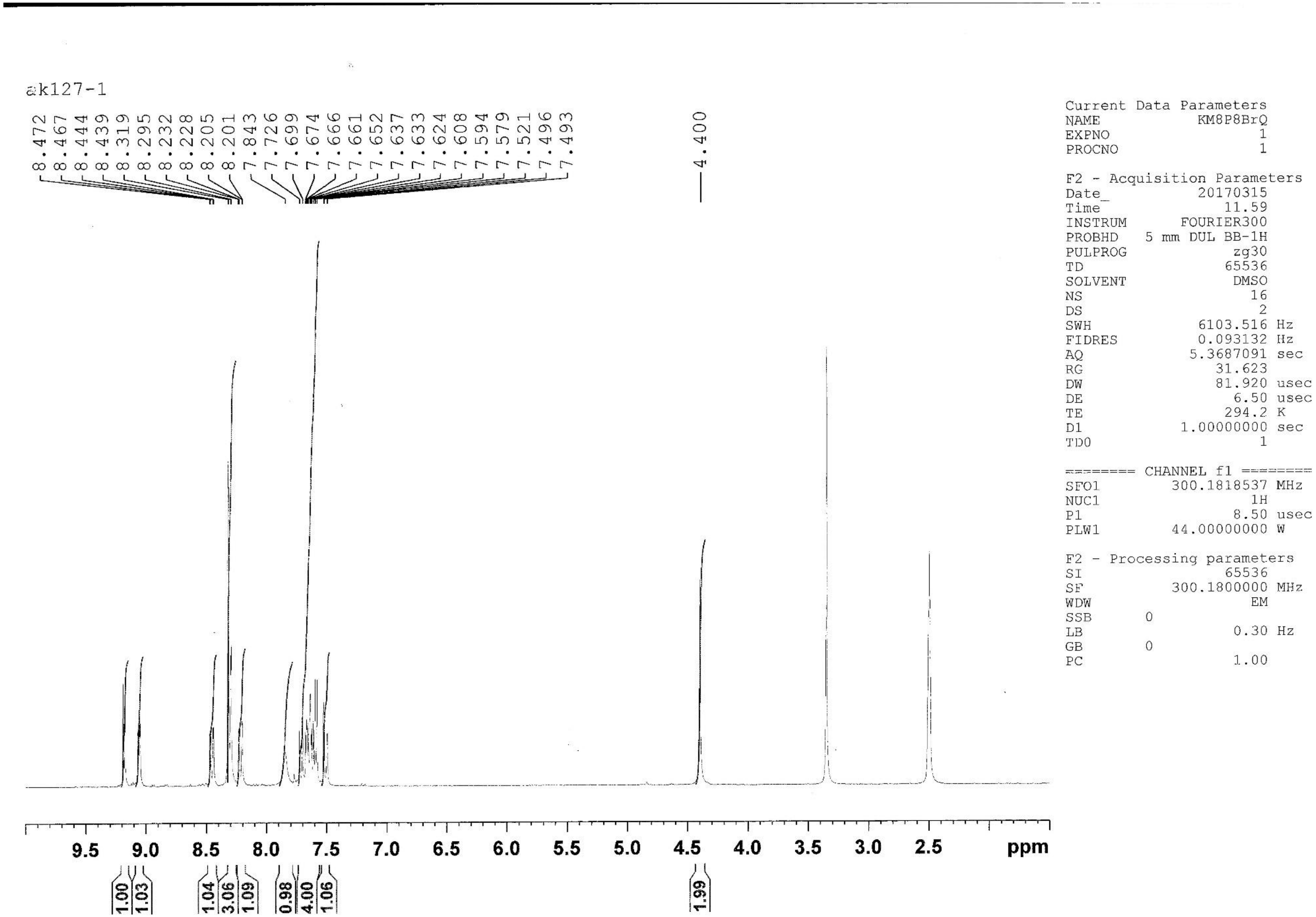

Figure 12. ^{1}H NMR spectra of compound **10a**

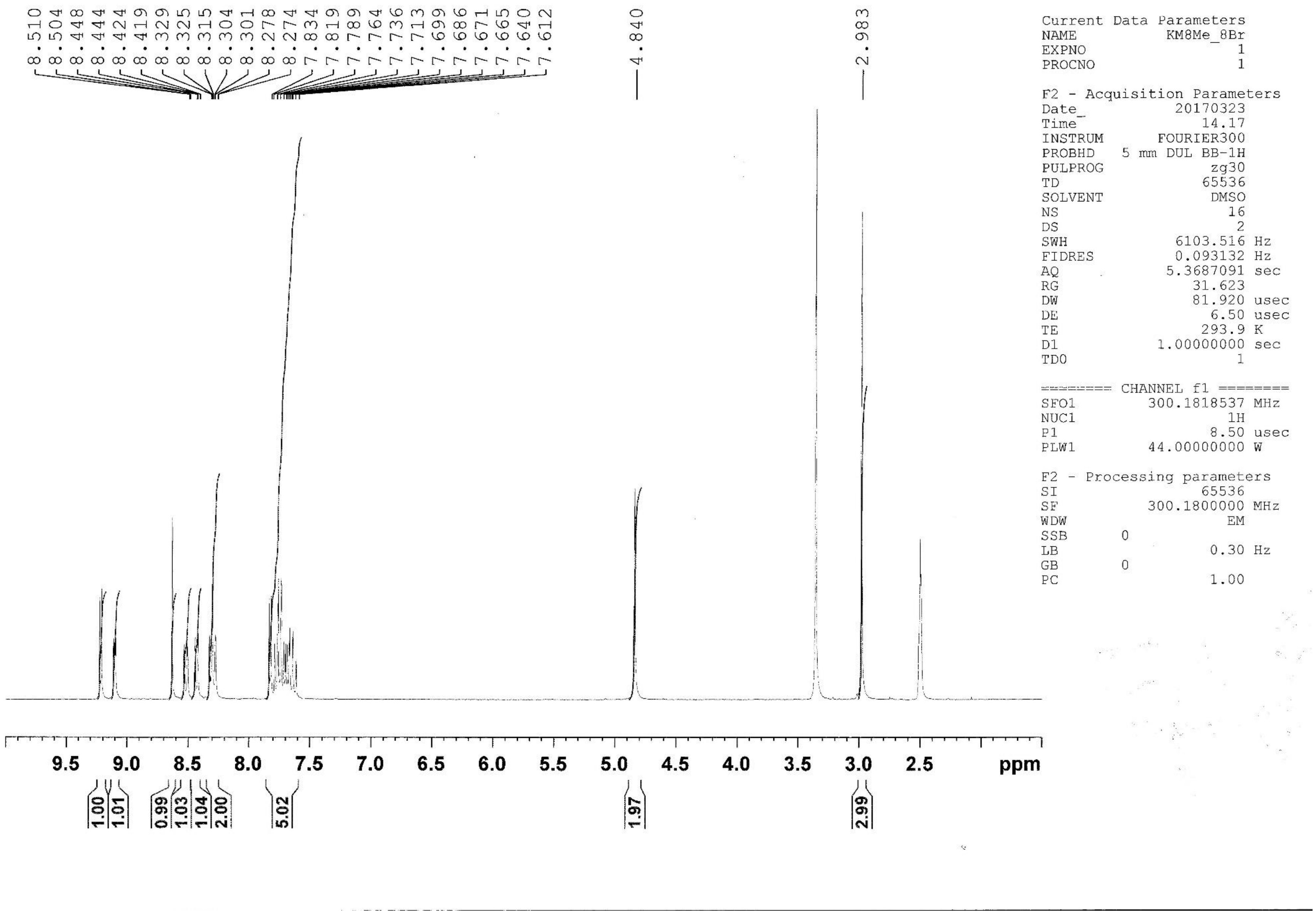

Figure 13. ^{1}H NMR spectra of compound **10b**

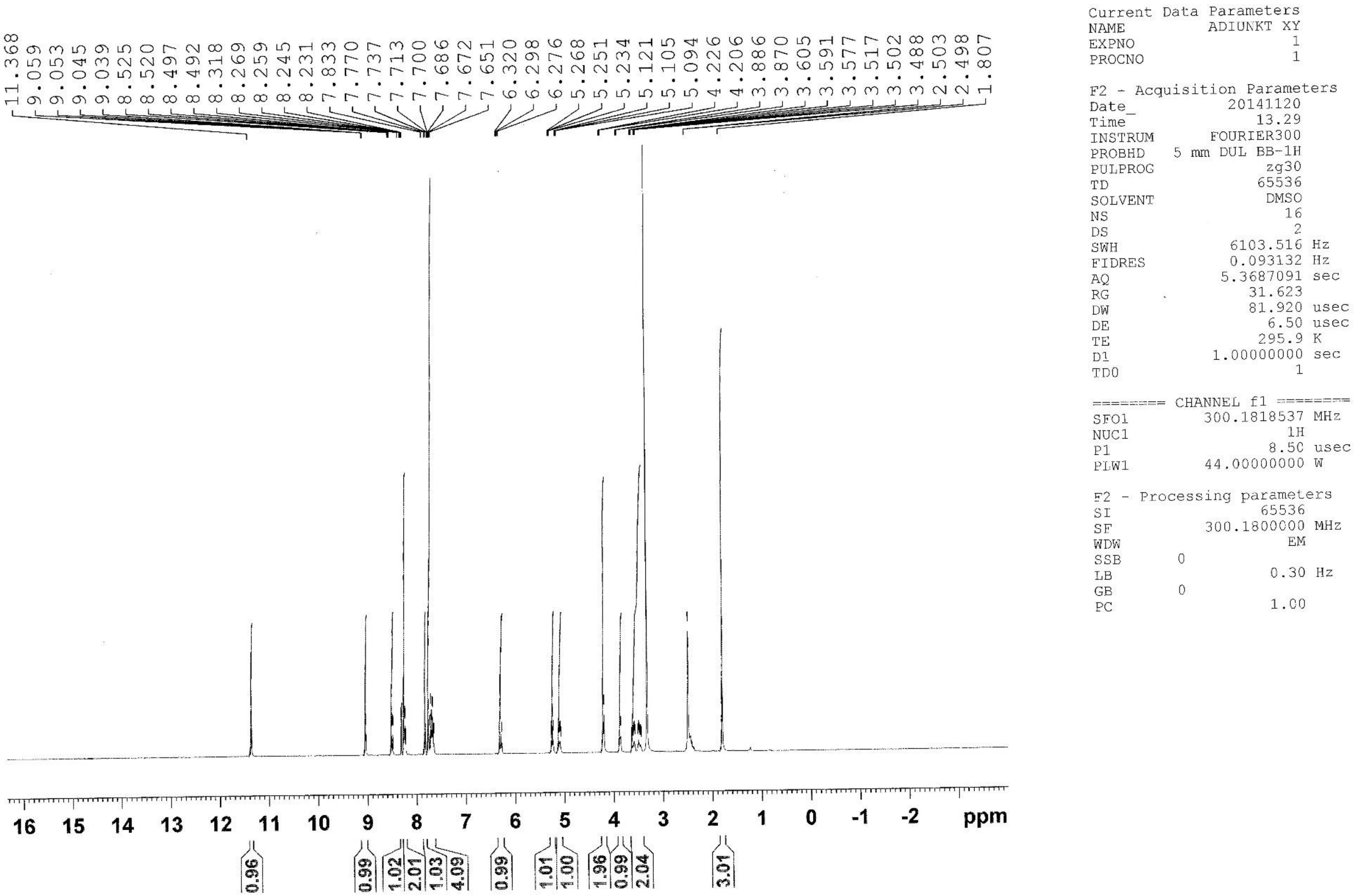

Figure 14. ^{1}H NMR spectra of compound **11a**

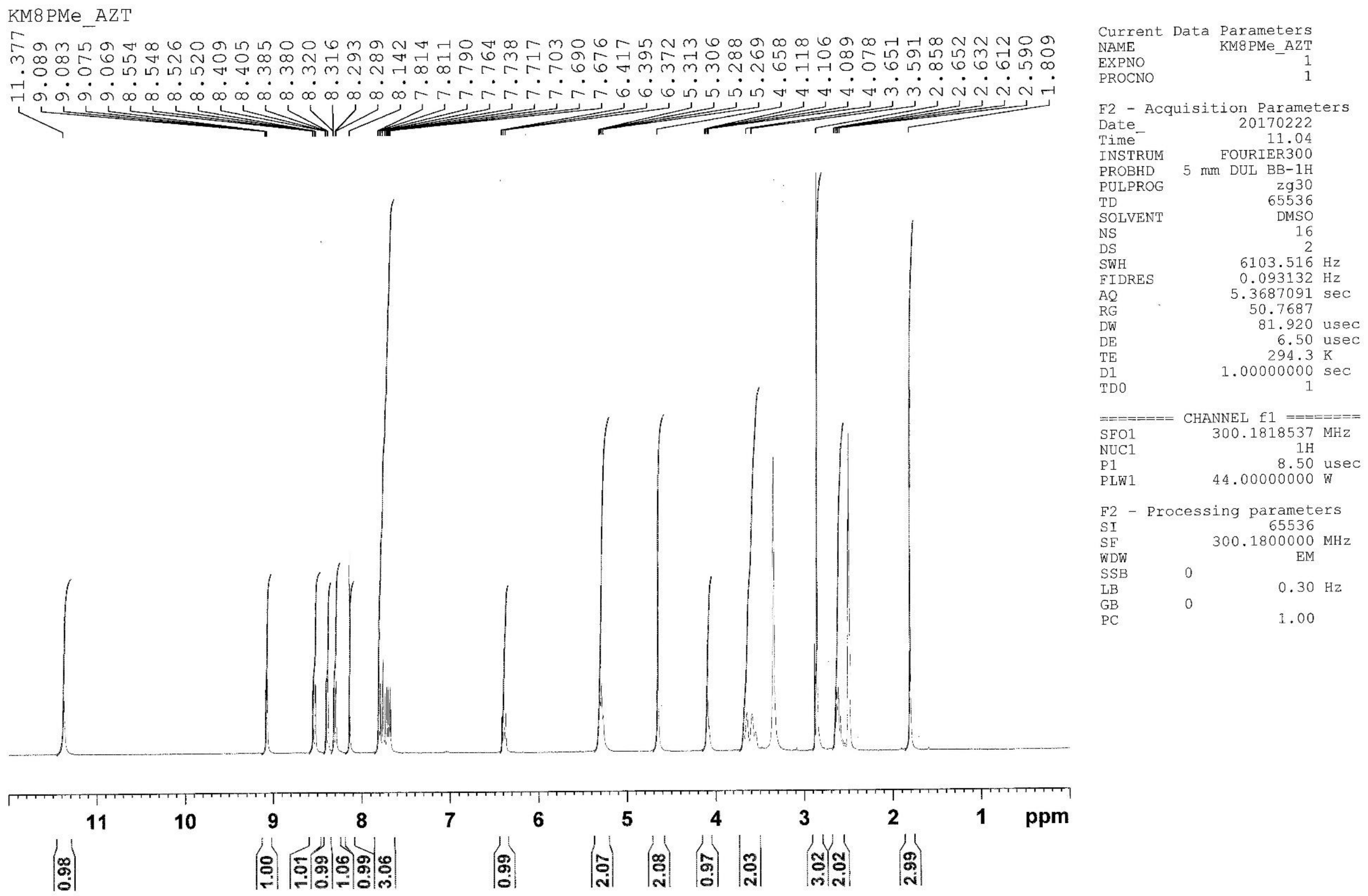

Figure 15. ^{1}H NMR spectra of compound **11b**

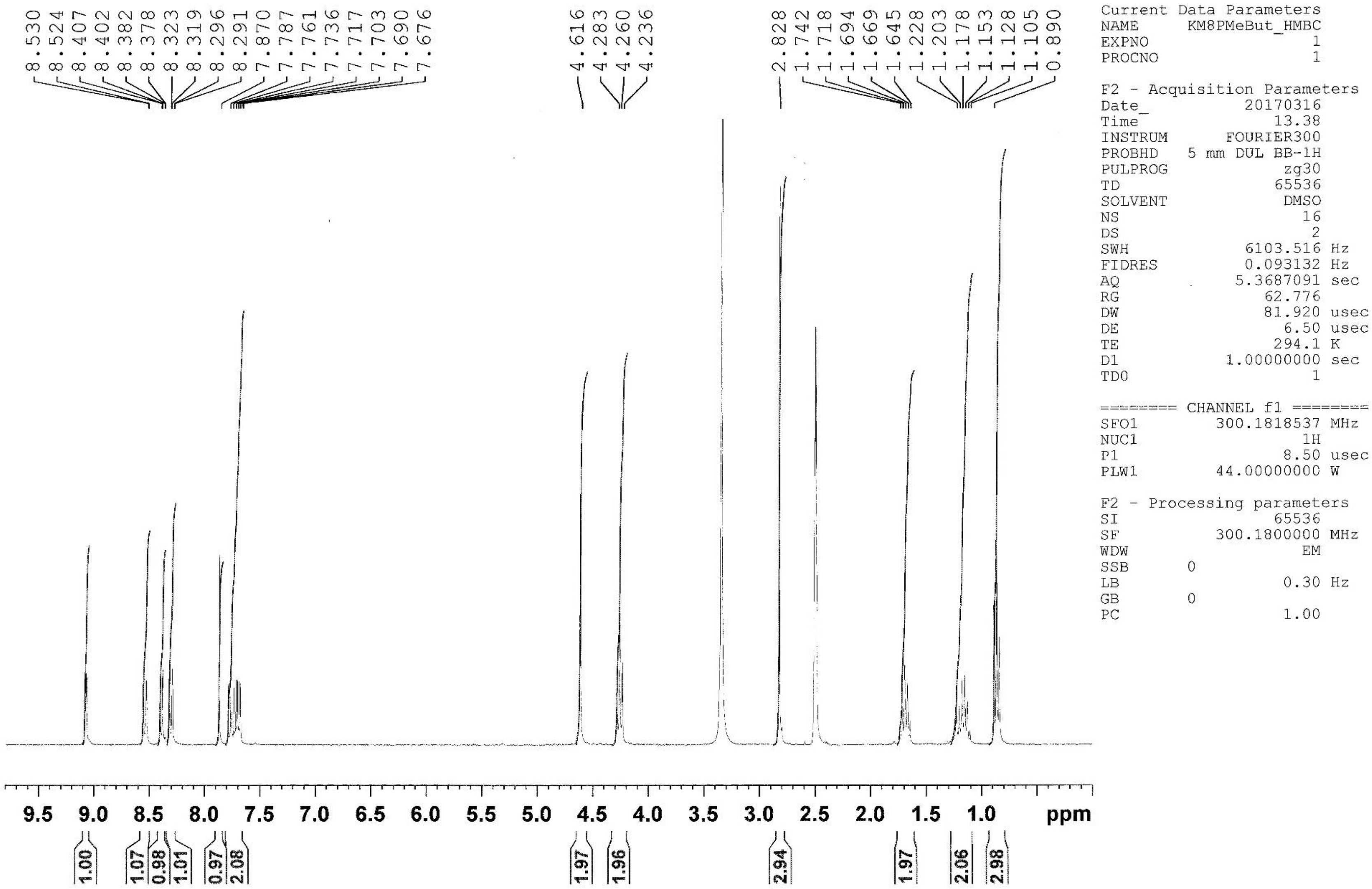

Figure 16. ^{1}H NMR spectra of compound **12b**

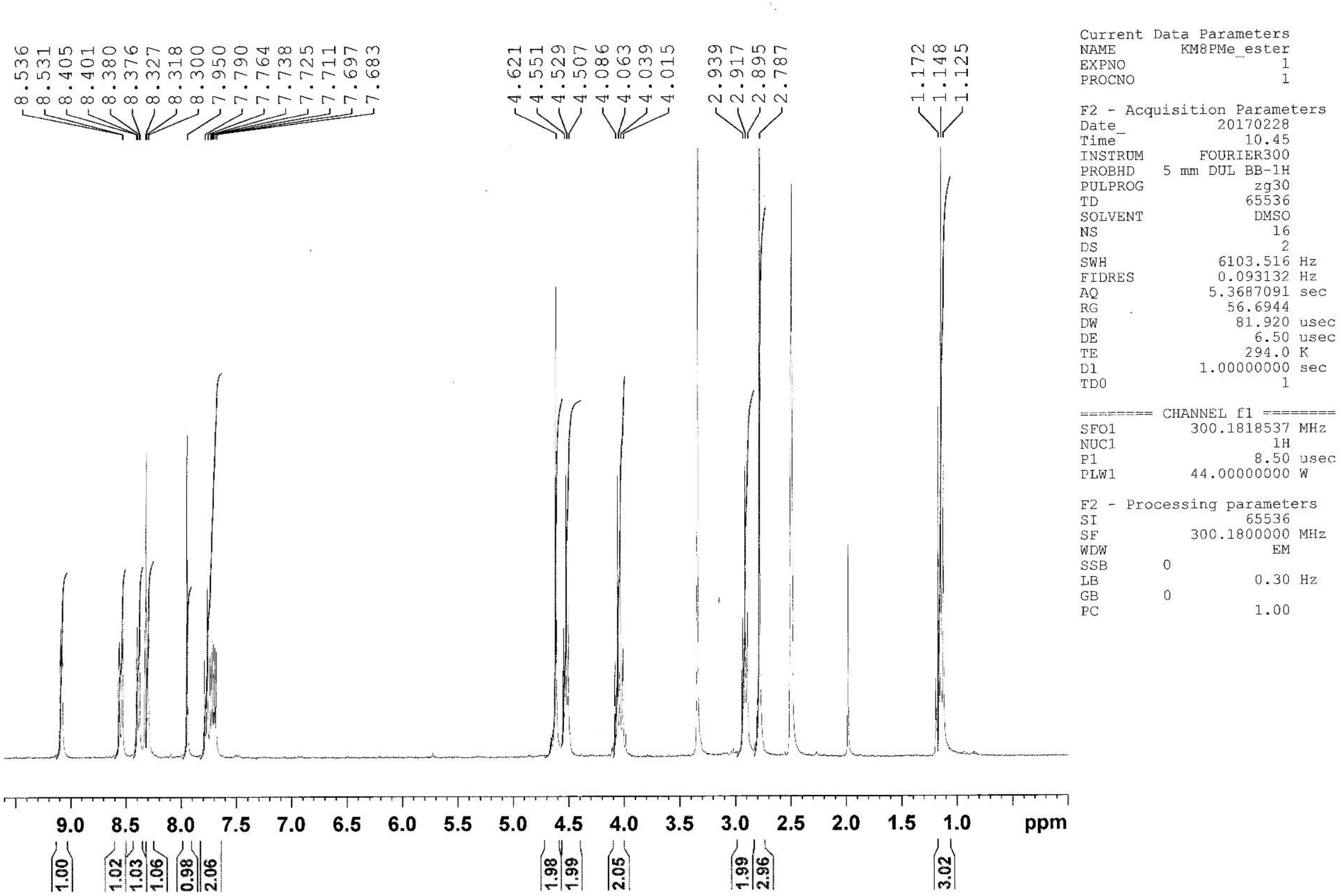

Figure 17. ^{1}H NMR spectra of compound **13b**

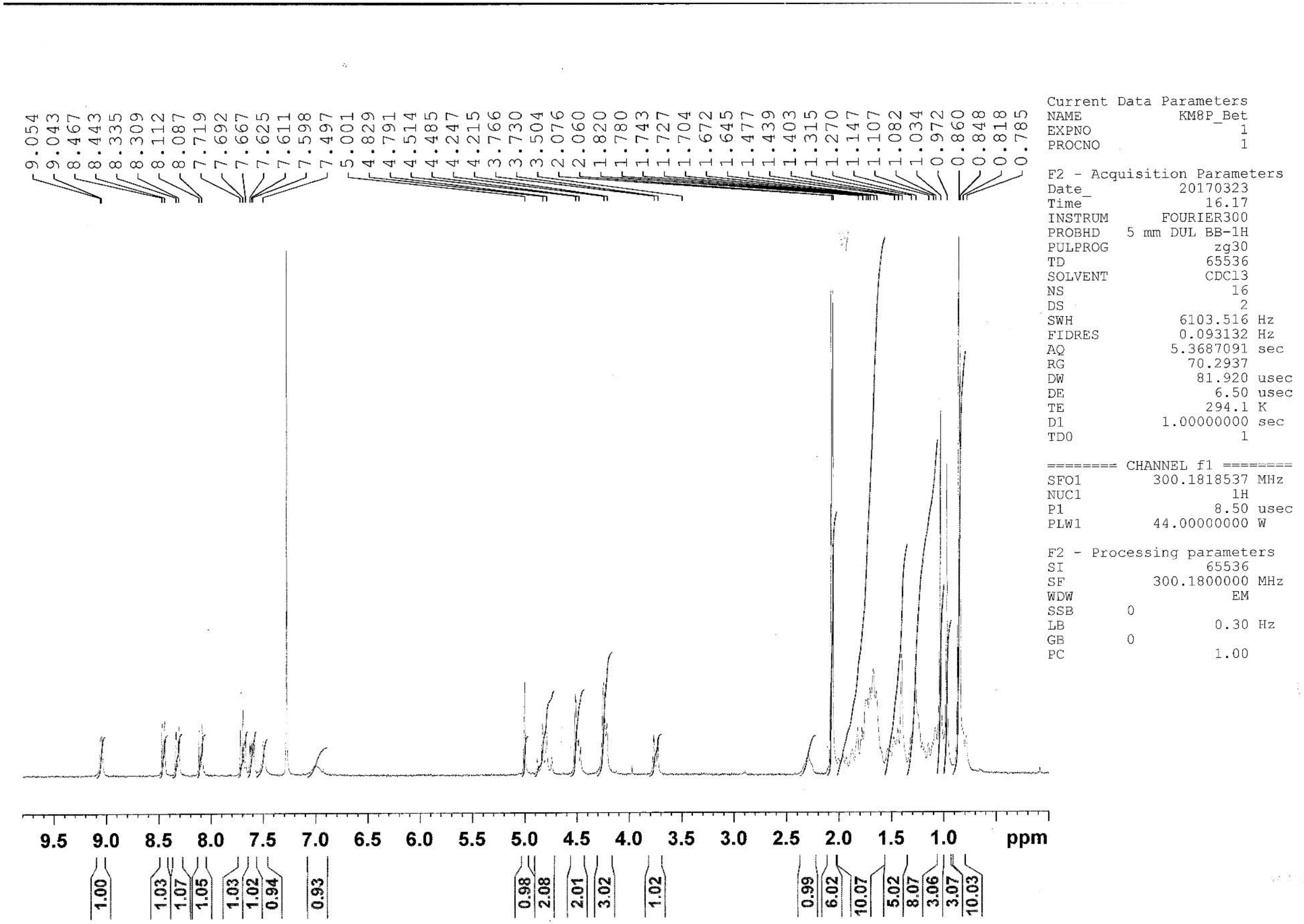

Figure 18. ^{1}H NMR spectra of compound **14a**

2. ^{13}C NMR spectra

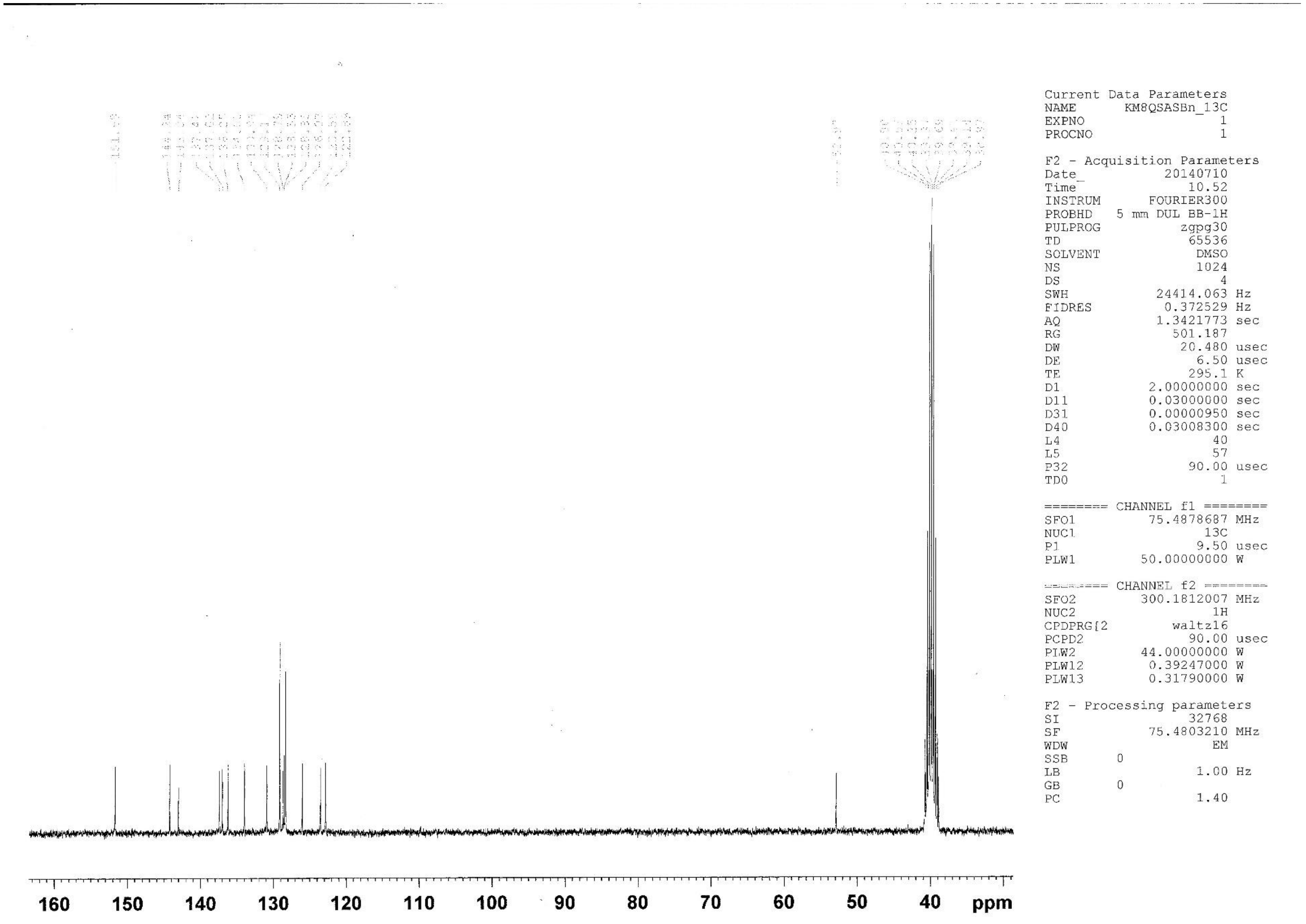

Figure 19. ^{13}C NMR spectra of compound **4a**

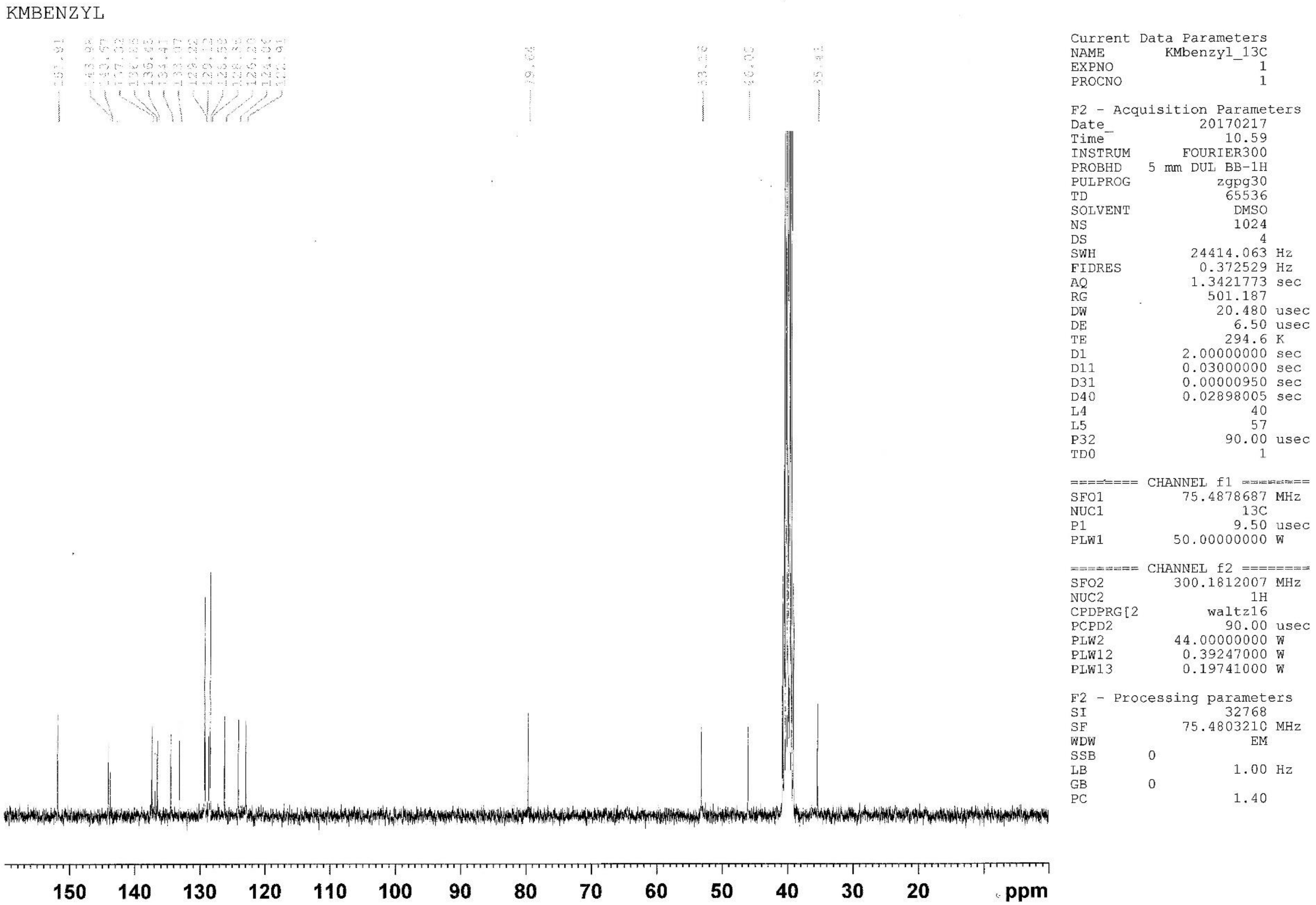

Figure 20. ^{13}C NMR spectra of compound **4b**

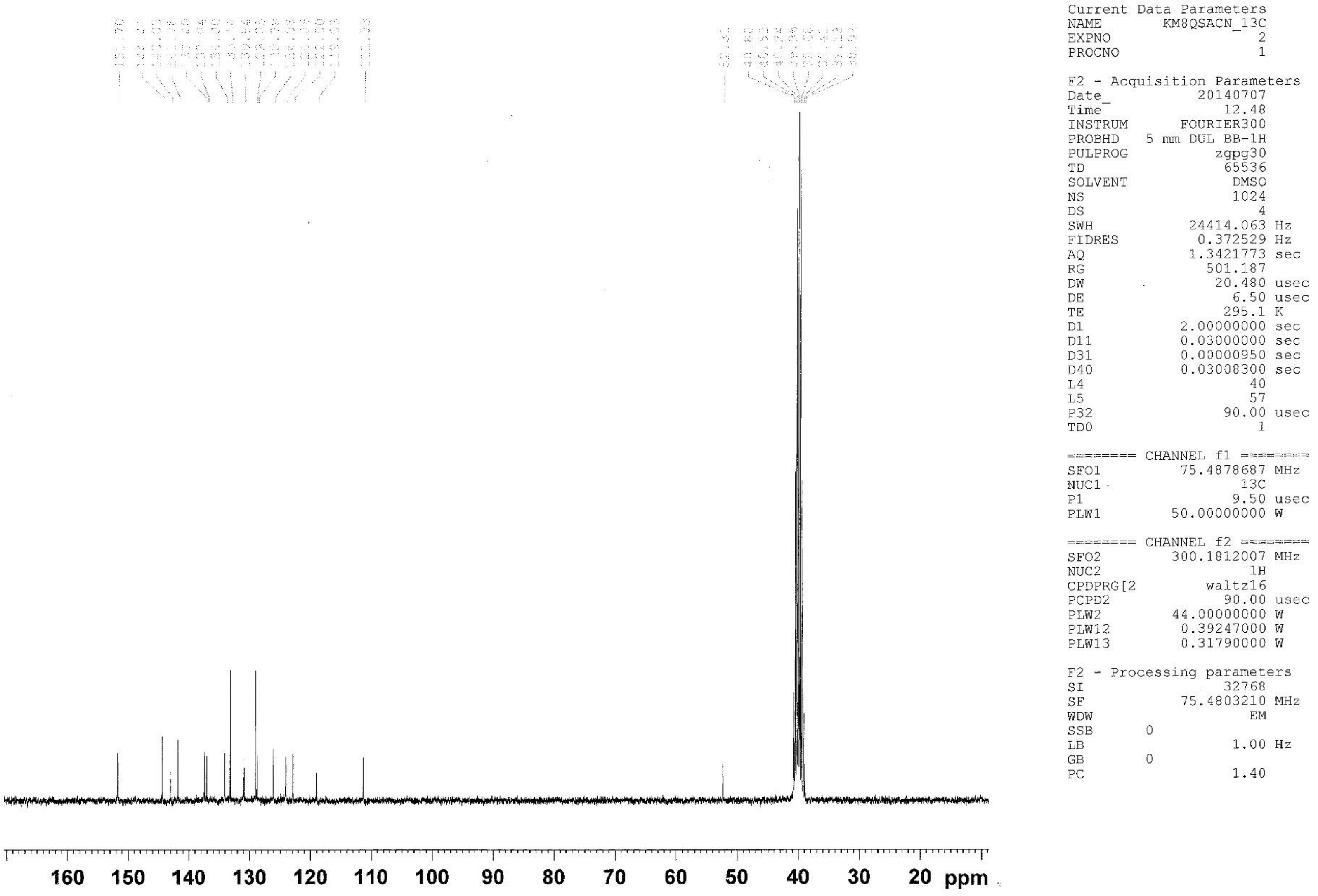

Figure 21. ^{13}C NMR spectra of compound **5a**

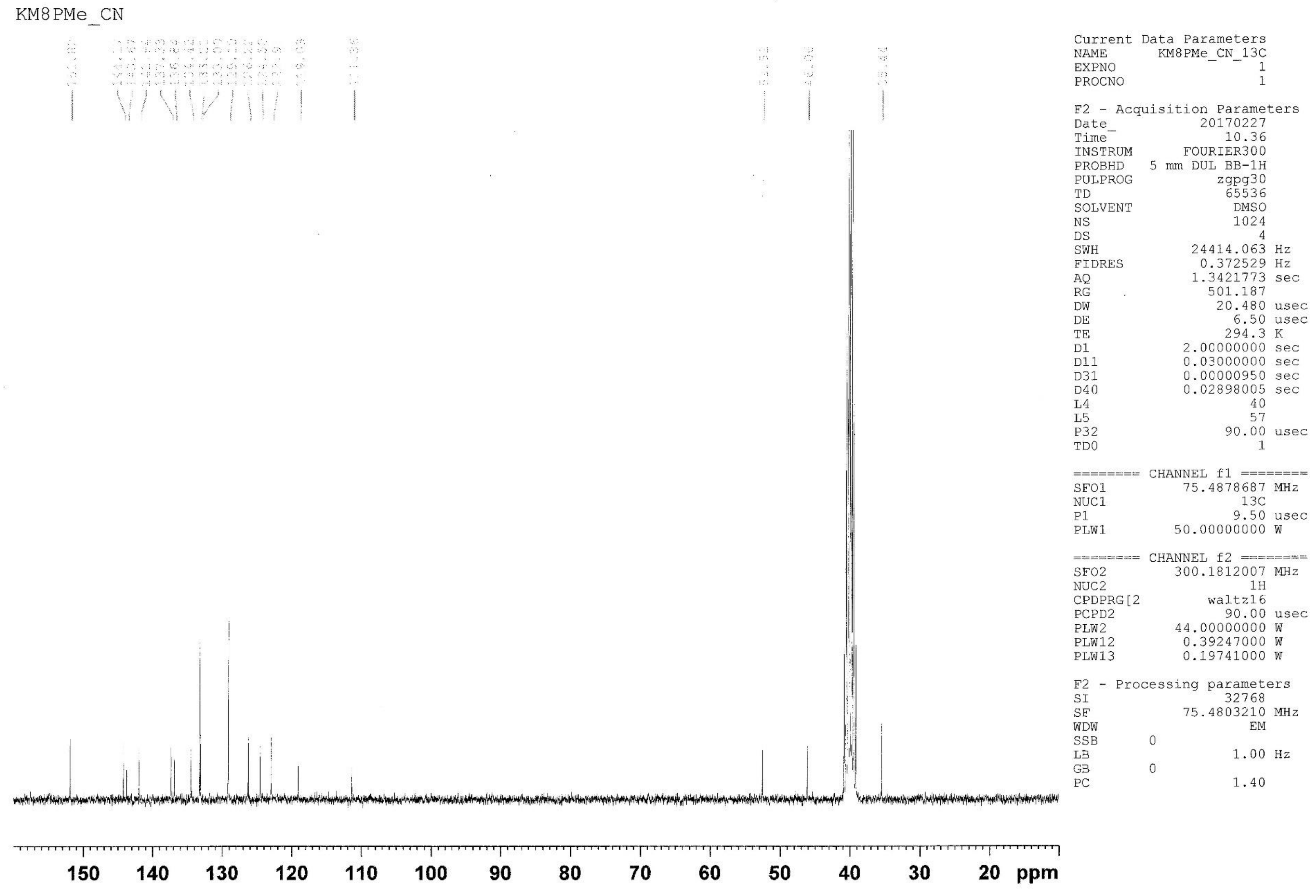

Figure 22. ^{13}C NMR spectra of compound **5b**

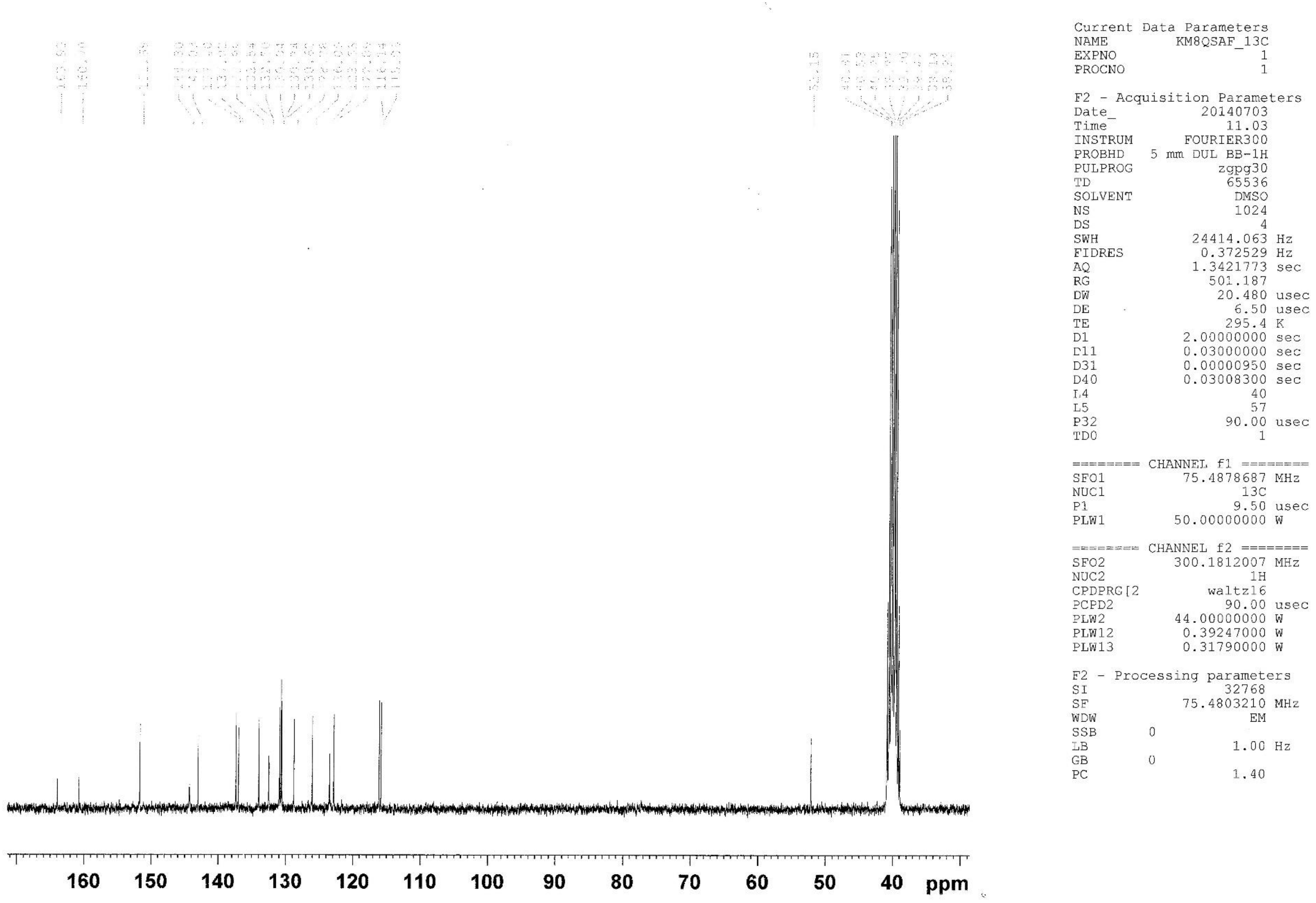

Figure 23. ^{13}C NMR spectra of compound **6a**

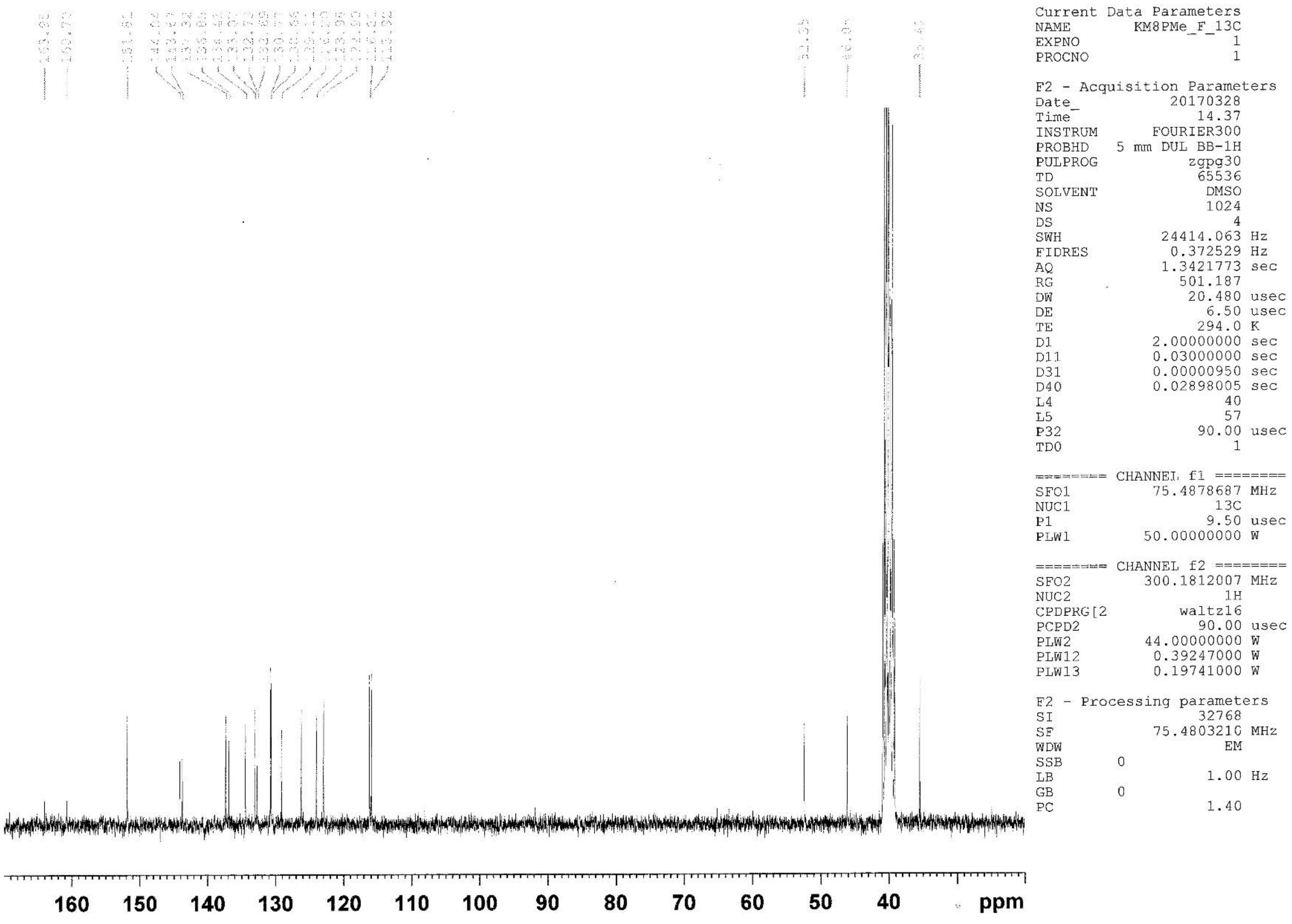

Figure 24. ^{13}C NMR spectra of compound **6b**

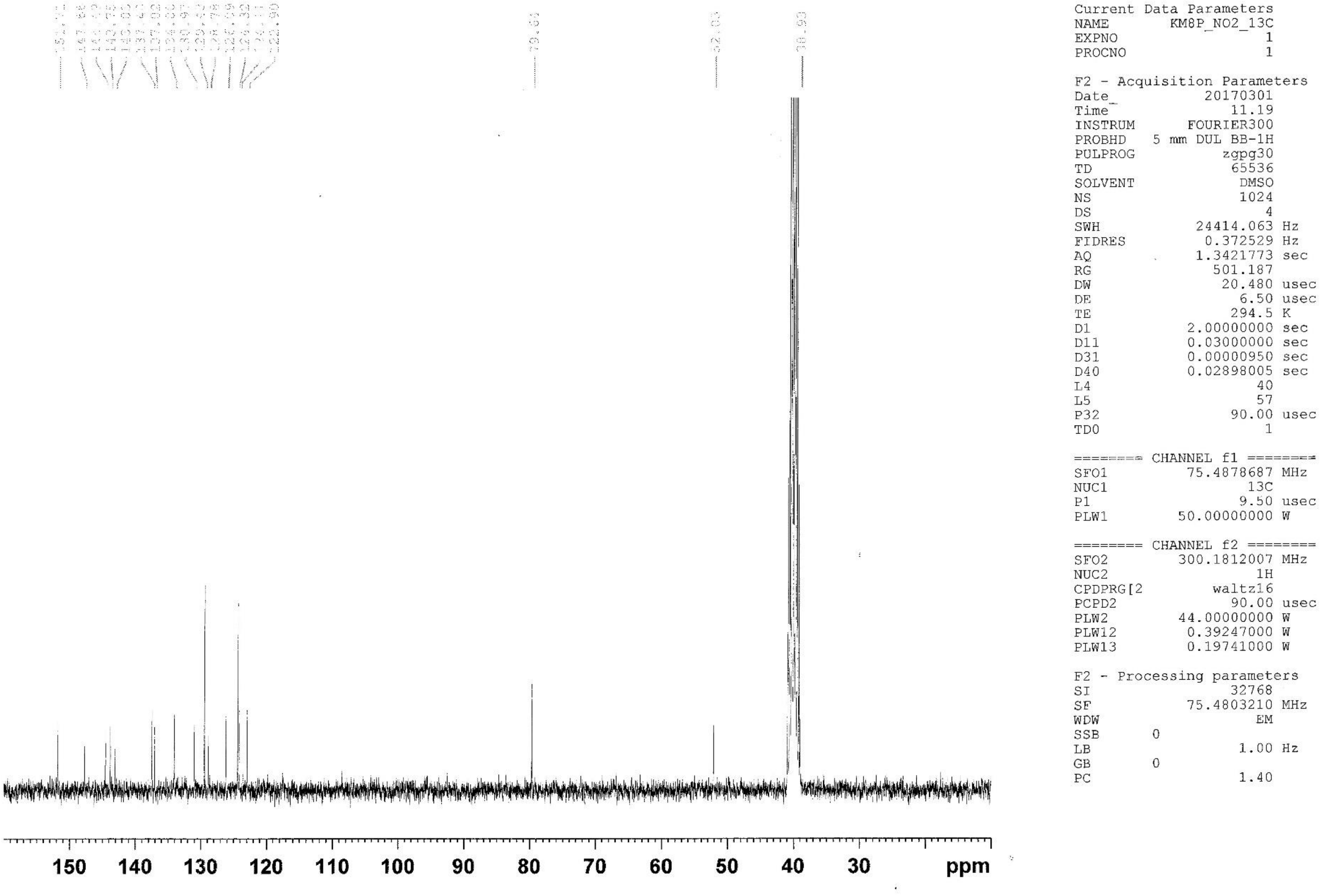

Figure 25. ^{13}C NMR spectra of compound **7a**

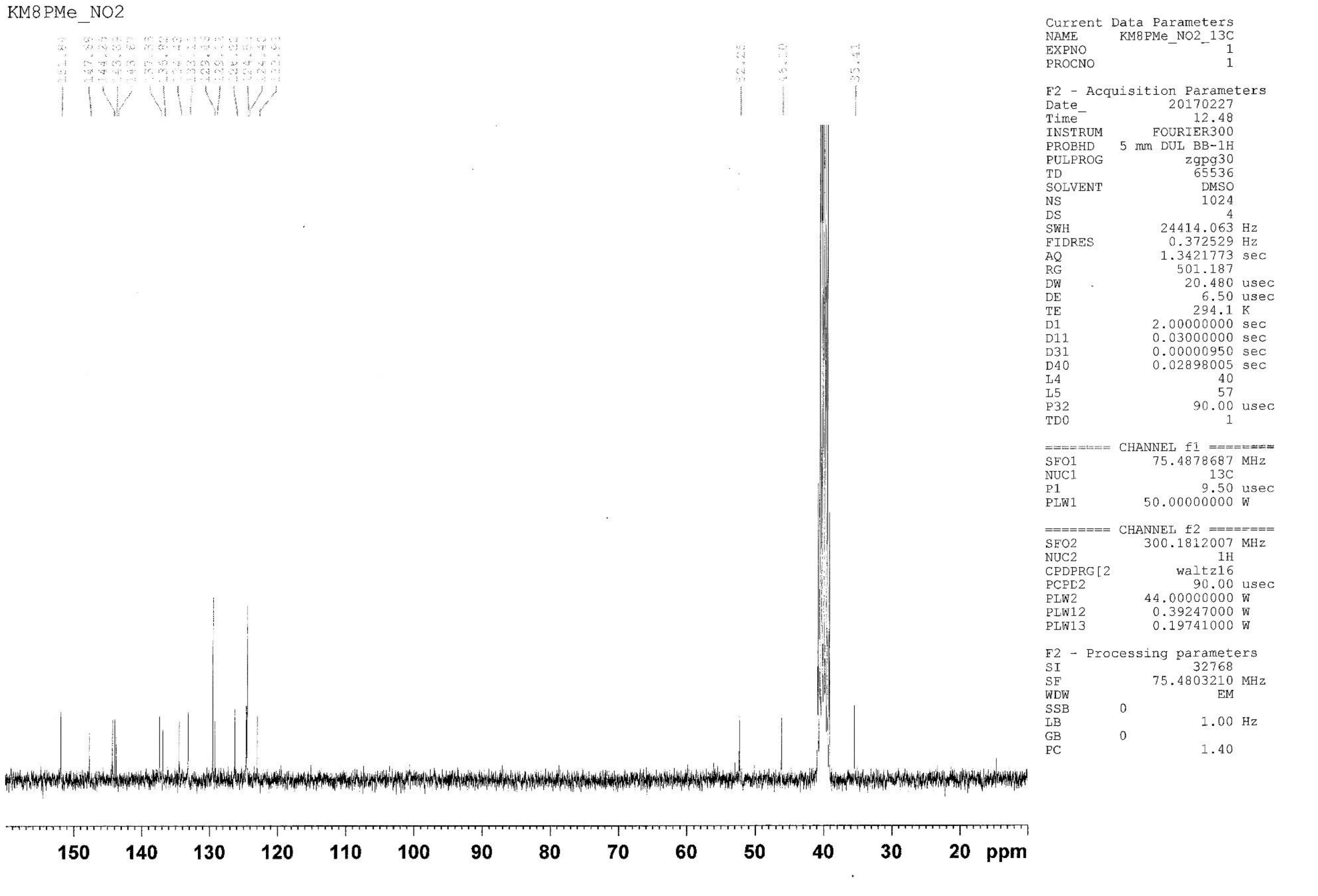

Figure 26. ^{13}C NMR spectra of compound **7b**

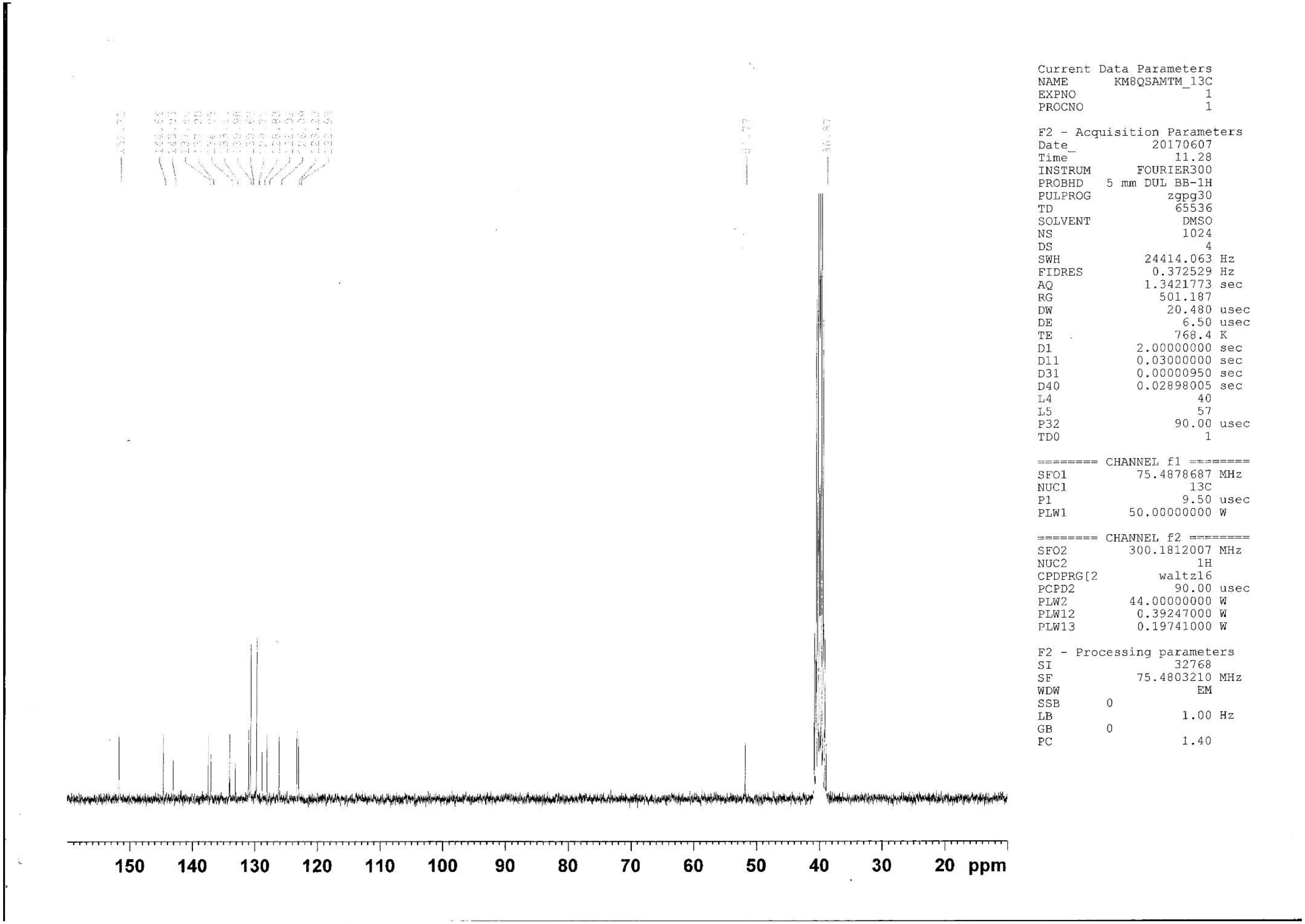

Figure 27. ^{13}C NMR spectra of compound **8a**

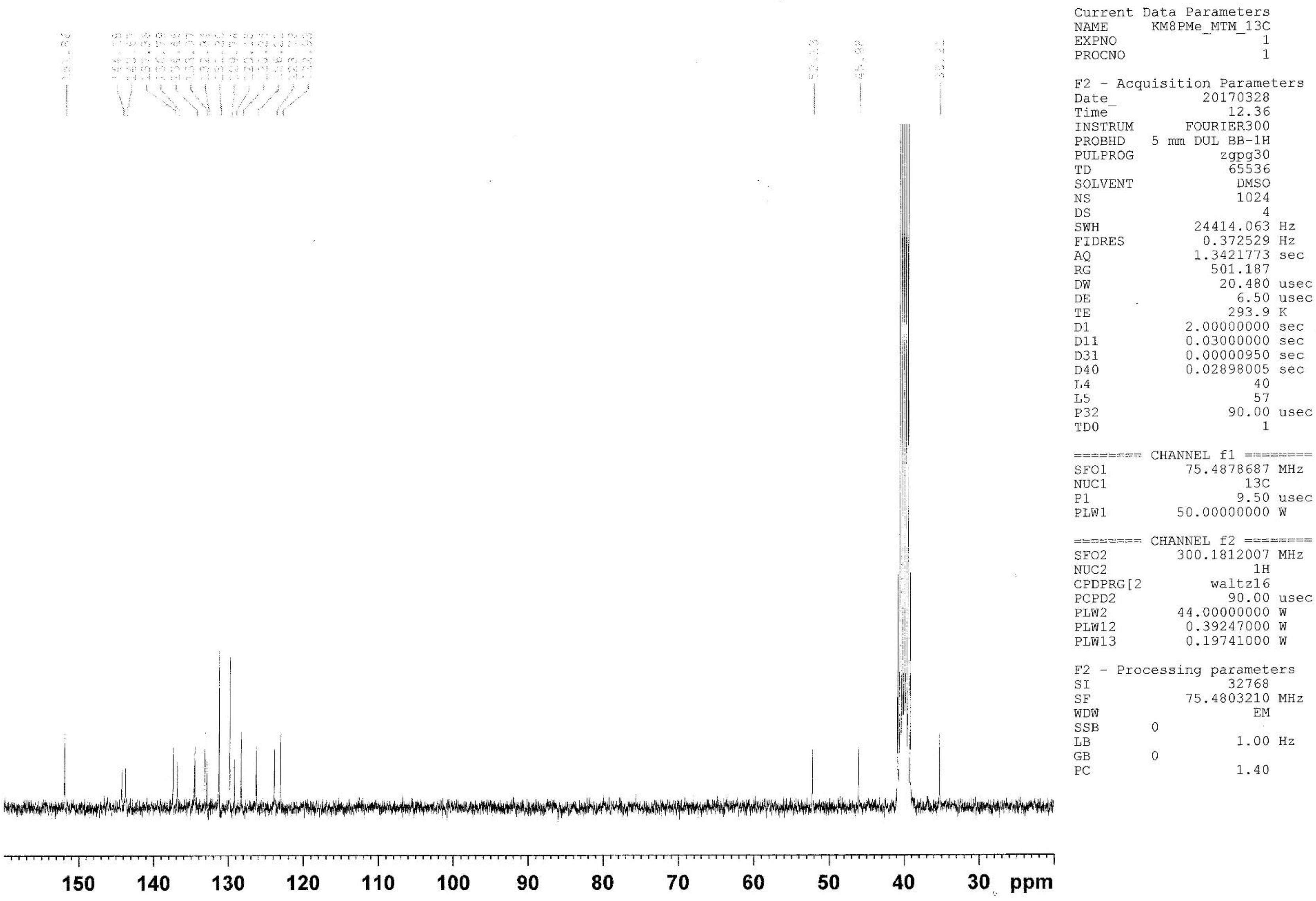

Figure 28. ^{13}C NMR spectra of compound **8b**

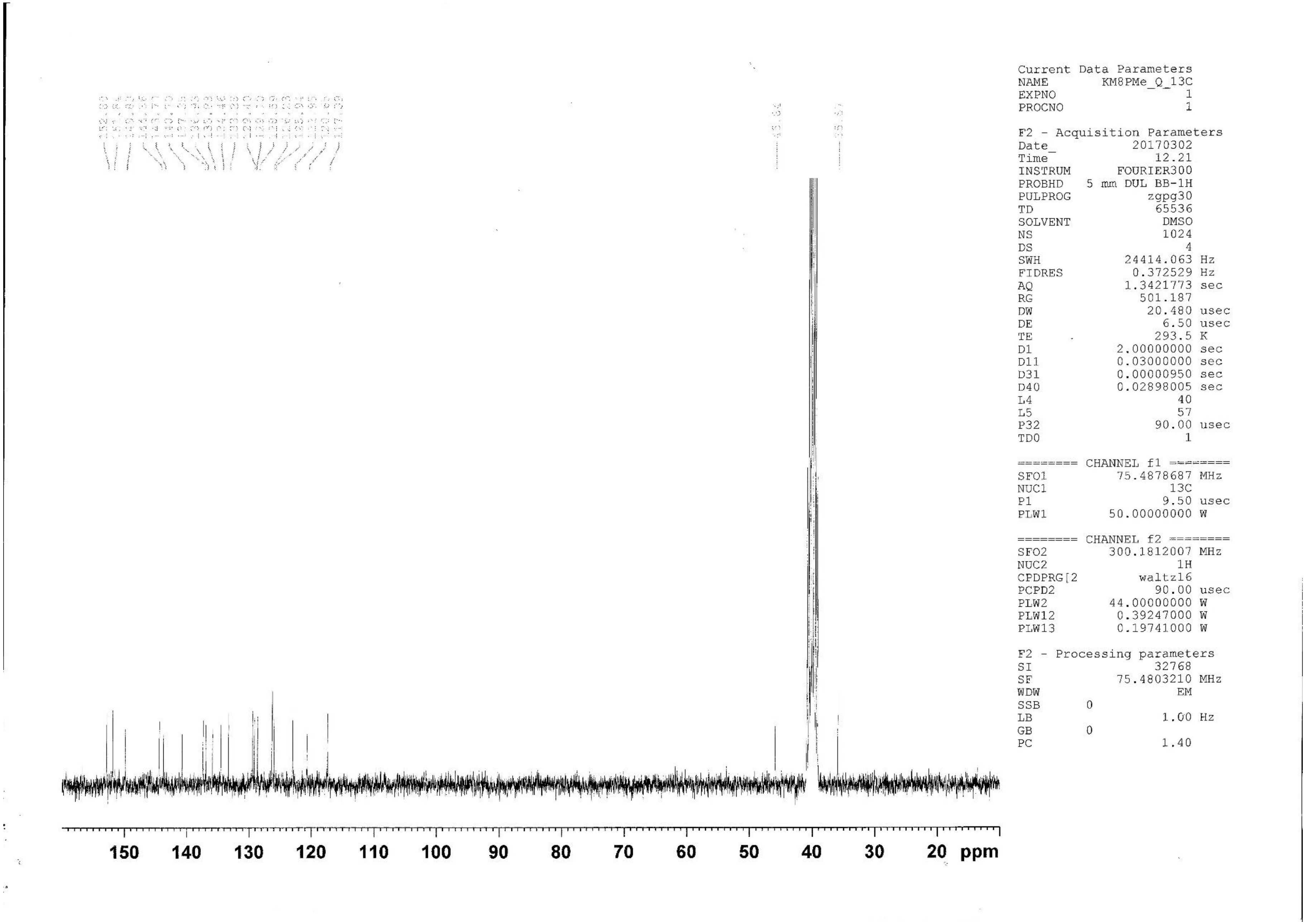

Figure 29. ^{13}C NMR spectra of compound **9b**

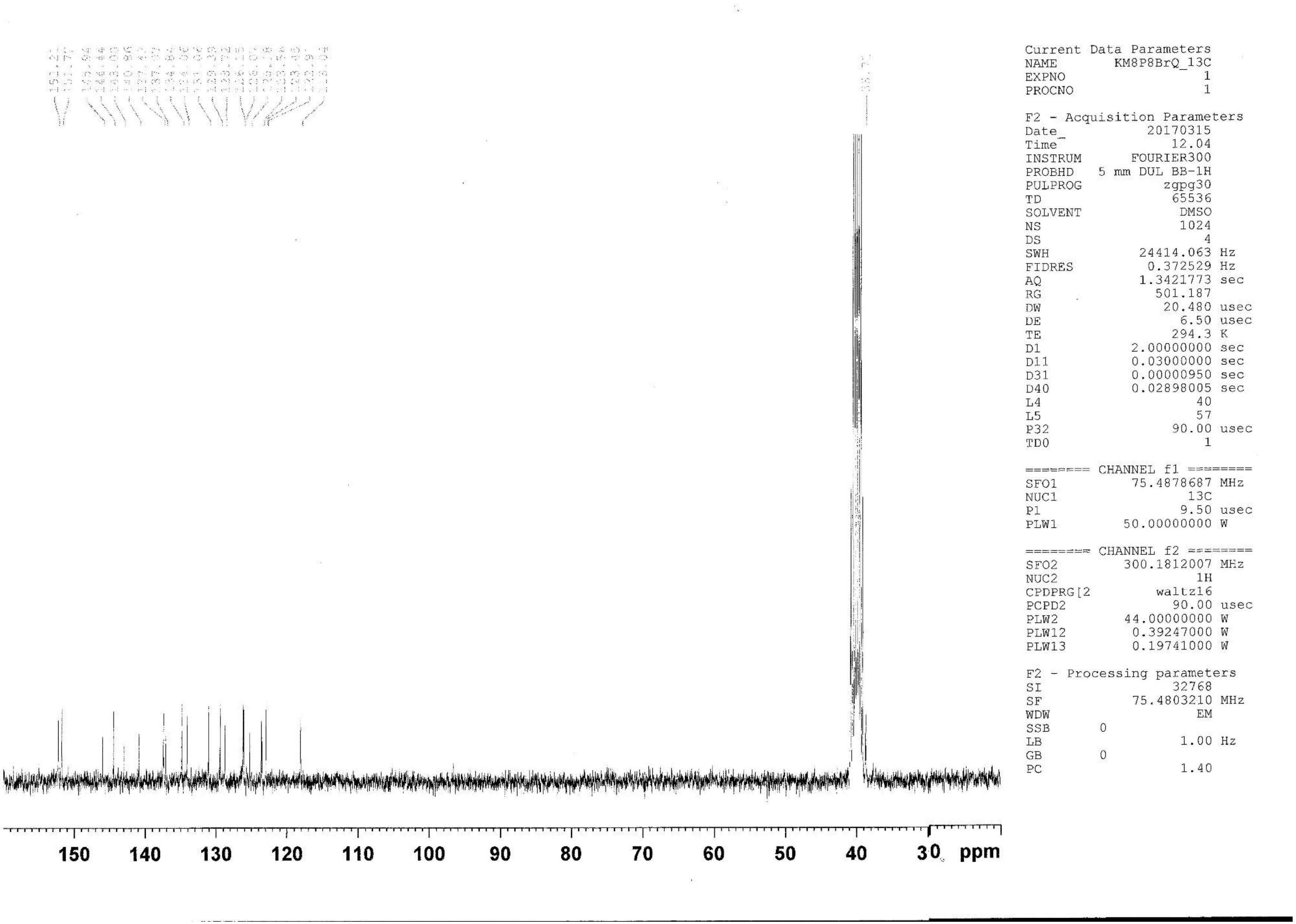

Figure 30. ^{13}C NMR spectra of compound **10a**

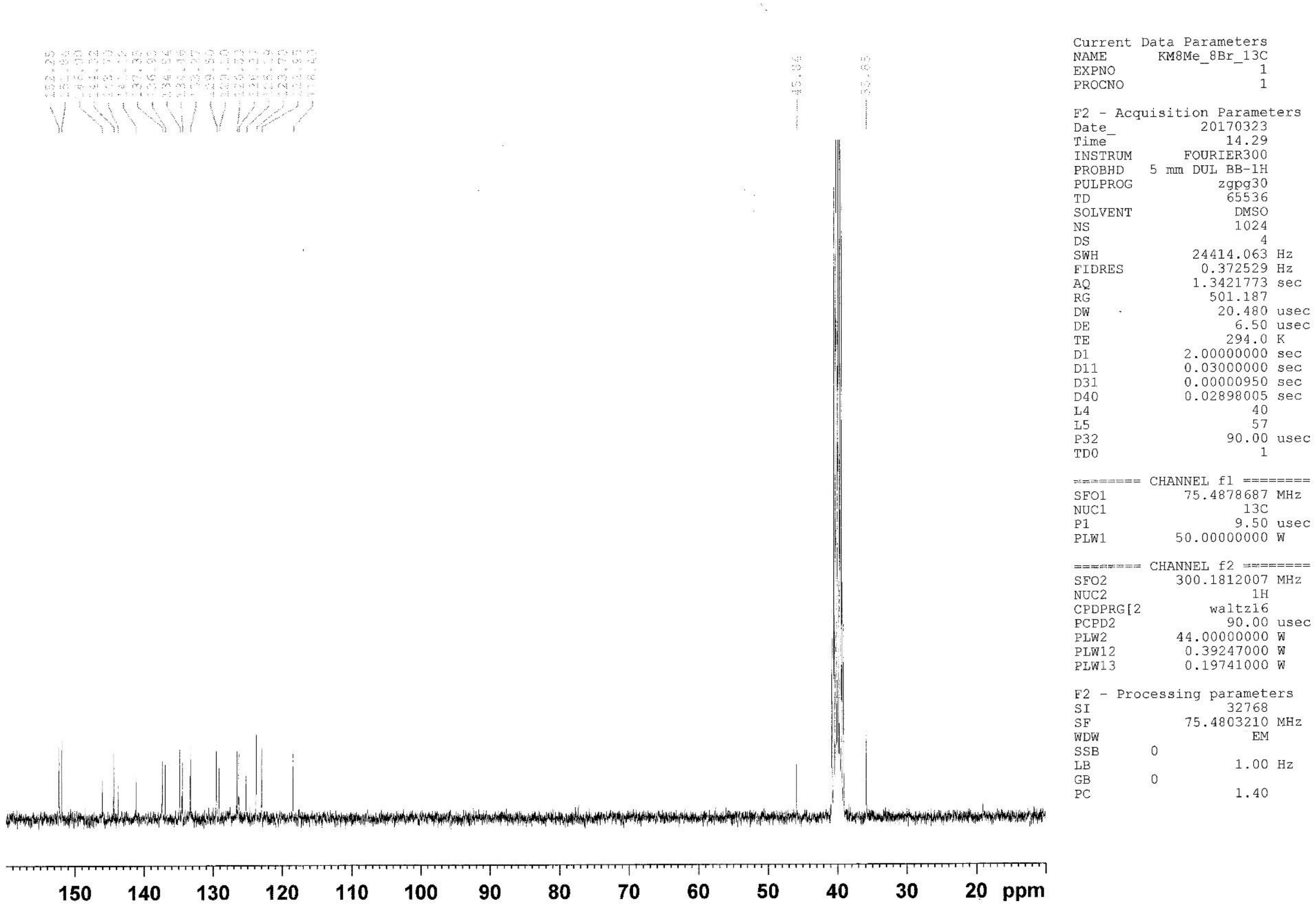

Figure 31. ^{13}C NMR spectra of compound **10b**

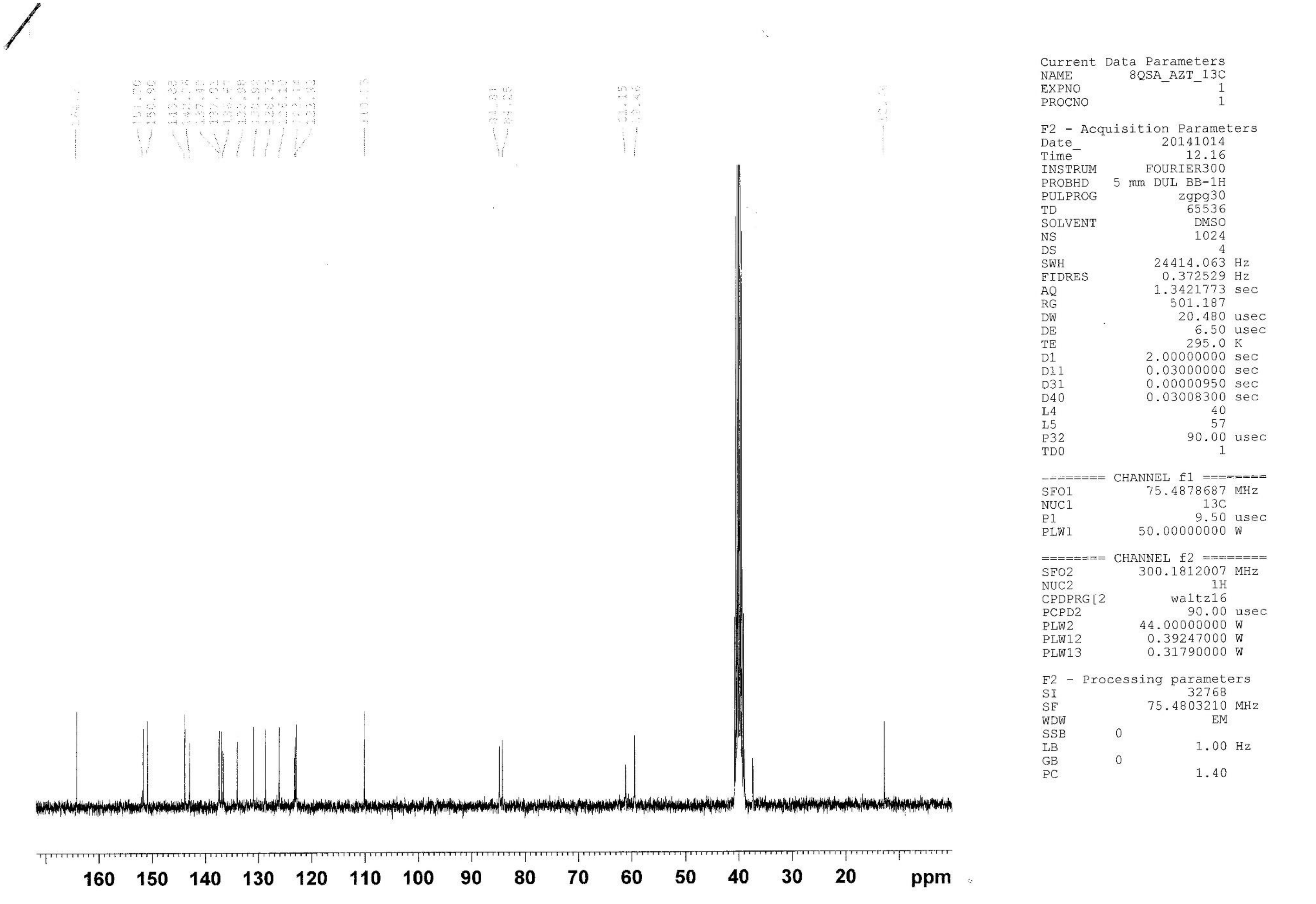

Figure 32. ^{13}C NMR spectra of compound **11a**

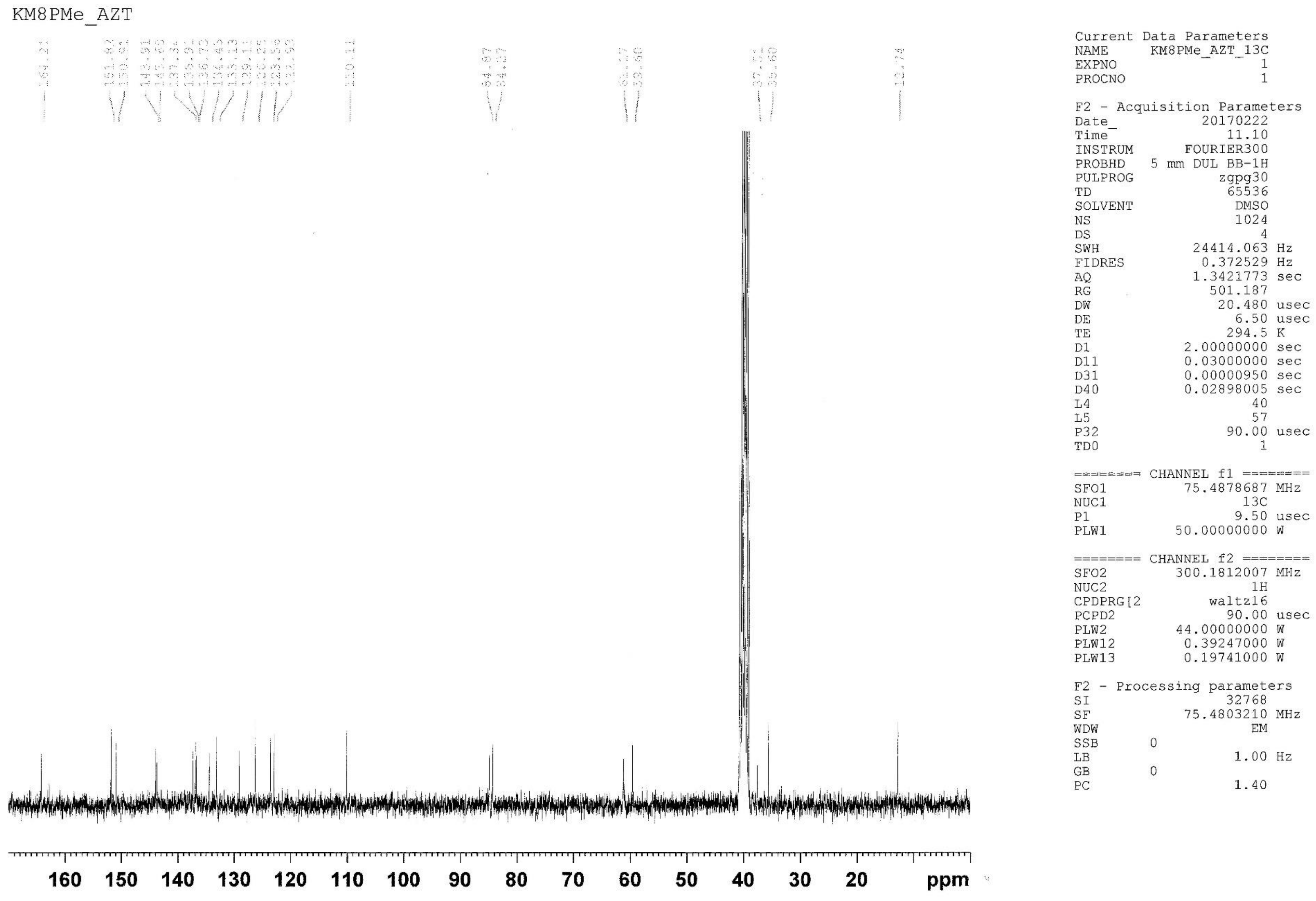

Figure 33. ^{13}C NMR spectra of compound **11b**

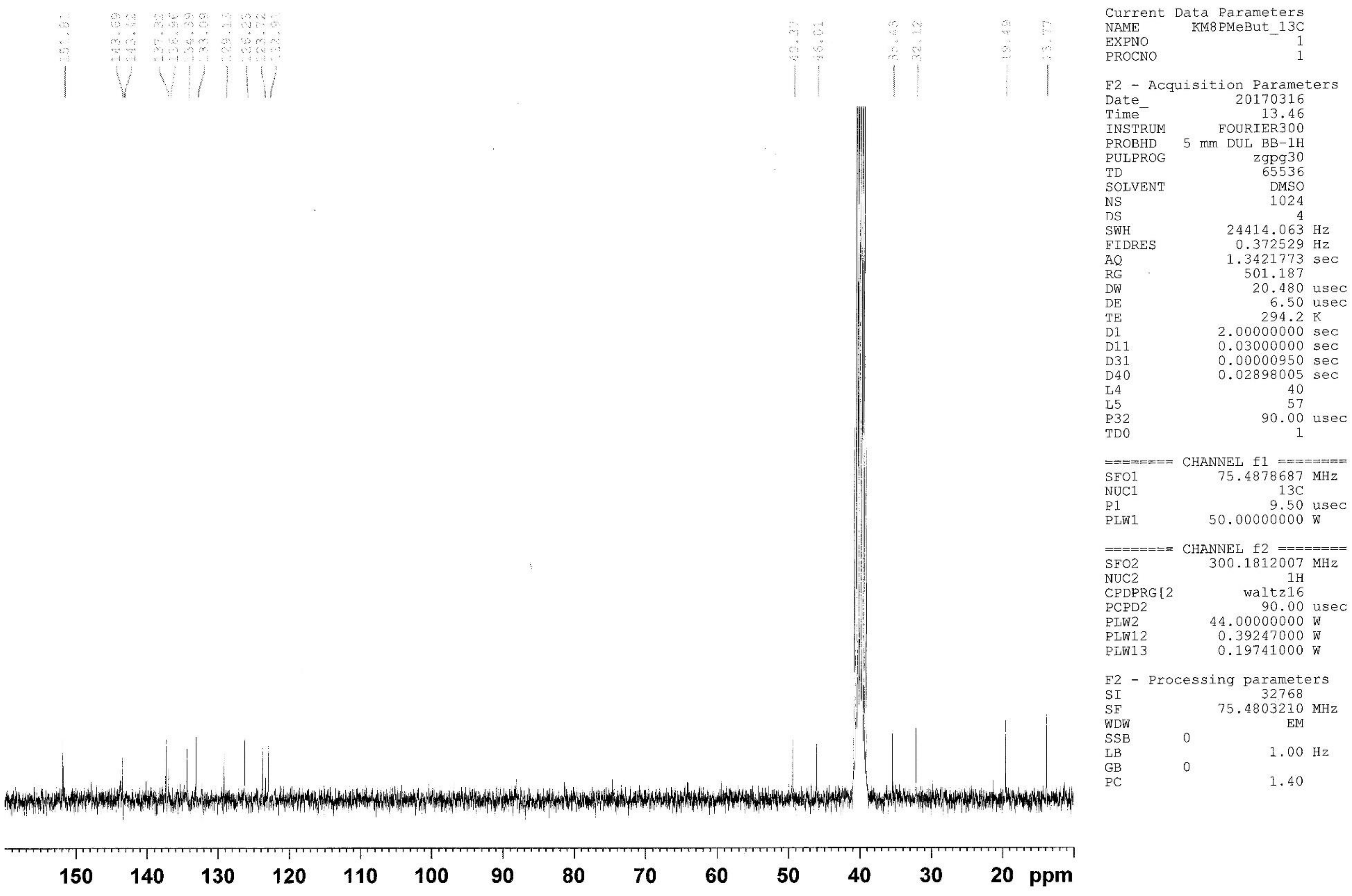

Figure 34. ^{13}C NMR spectra of compound **12b**

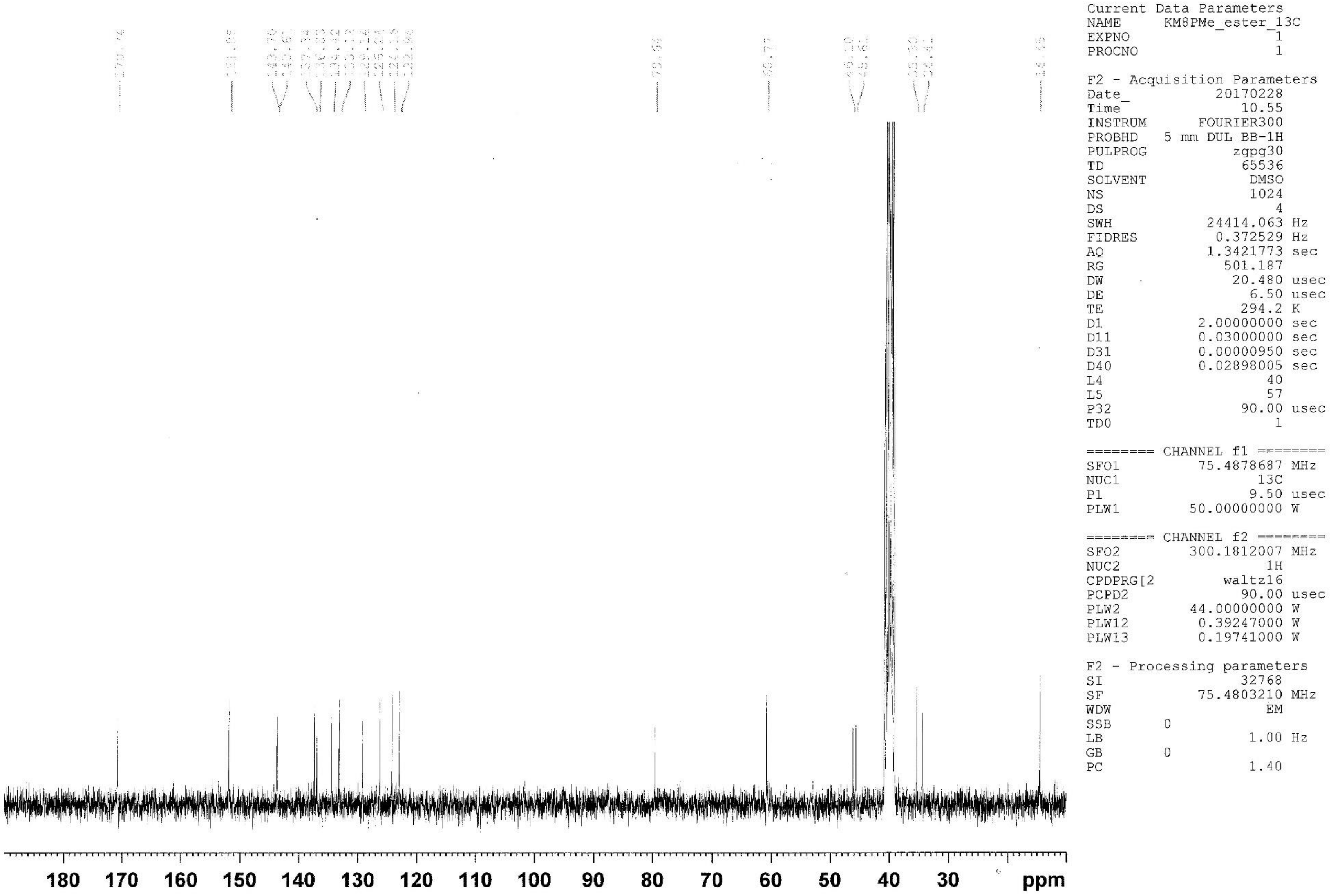

Figure 35. ^{13}C NMR spectra of compound **13b**

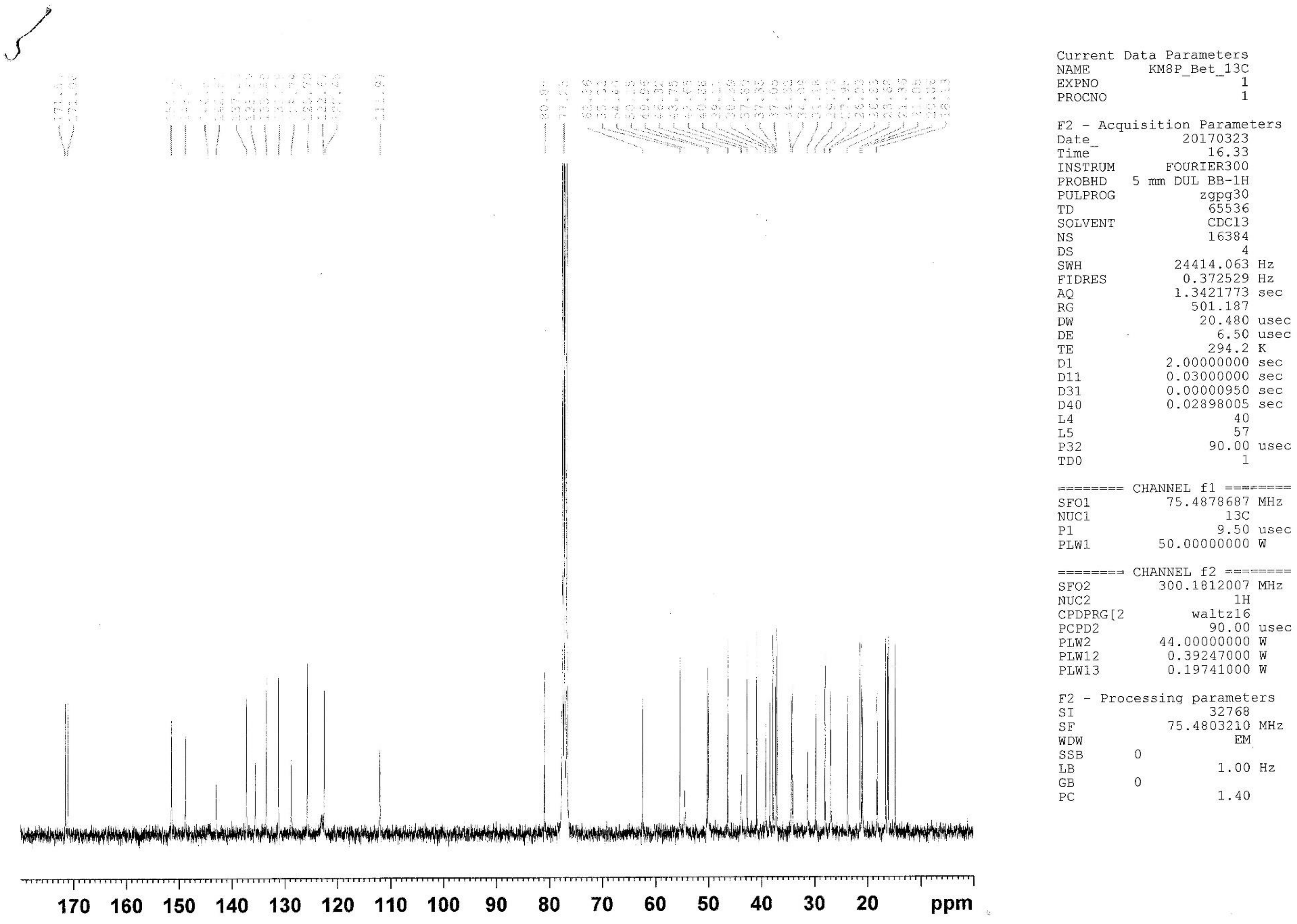

Figure 36. ^{13}C NMR spectra of compound **14a**

3. HR MS spectra

Compound Spectrum List Report

Analysis Info

Analysis Name	D:\Data\KMBenzyl.d	Acquisition Date	6/5/2017 1:14:11 PM
Method	low_mass.m	Operator	KM
Sample Name	TM Low concentration	Instrument	impact II 1825265.10082
Comment			

Acquisition Parameter

Source Type	ESI	Ion Polarity	Positive	Set Nebulizer	0.3 Bar
Focus	Active	Set Capillary	4000 V	Set Dry Heater	240 °C
Scan Begin	100 m/z	Set End Plate Offset	-500 V	Set Dry Gas	4.0 l/min
Scan End	1000 m/z	Set Charging Voltage	2000 V	Set Divert Valve	Source
		Set Corona	0 nA	Set APCI Heater	0 °C

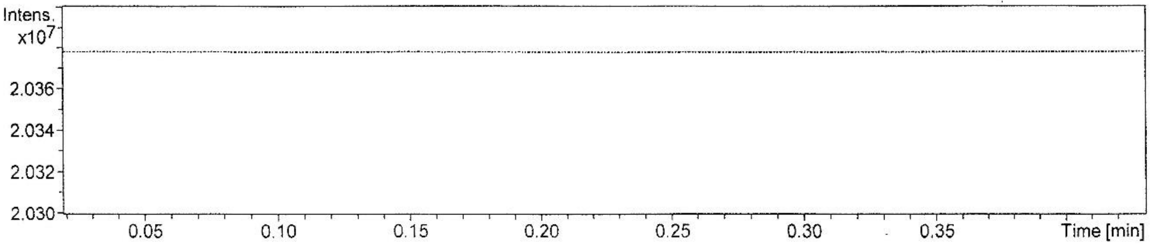

+MS, 0.0-0.4min #1-25

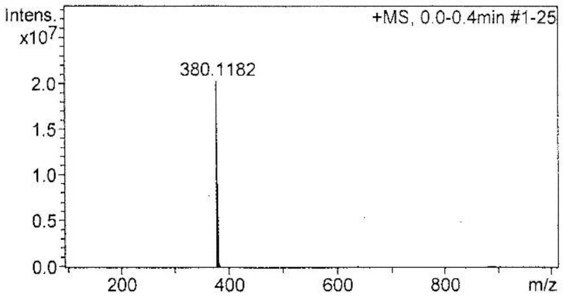

#	m/z	Res.	S/N	I	I %	FWHM
1	380.1182	19960	69230.5	20377580	100.0	0.0190

KMBenzyl.d

Bruker Compass DataAnalysis 4.3 printed: 6/5/2017 1:15:07 PM by: KM Page 1 of 1

4.

Figure 37. MS spectra of compound **4a**

Compound Spectrum List Report

Analysis Info

Analysis Name	D:\Data\KMMeBenzyl.d	Acquisition Date	6/5/2017 11:14:43 AM
Method	low_mass.m	Operator	KM
Sample Name	TM Low concentration	Instrument	impact II 1825265.10082
Comment			

Acquisition Parameter

Source Type	ESI	Ion Polarity	Positive	Set Nebulizer	0.3 Bar
Focus	Active	Set Capillary	4000 V	Set Dry Heater	240 °C
Scan Begin	100 m/z	Set End Plate Offset	-500 V	Set Dry Gas	4.0 l/min
Scan End	1000 m/z	Set Charging Voltage	2000 V	Set Divert Valve	Source
		Set Corona	0 nA	Set APCI Heater	0 °C

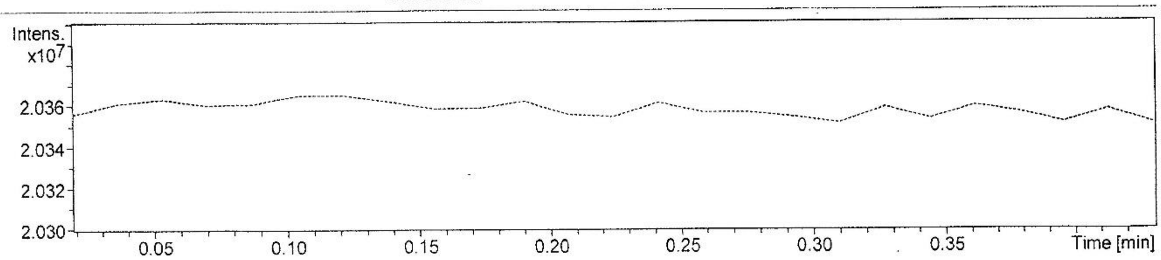

+MS, 0.0-0.4min #1-25

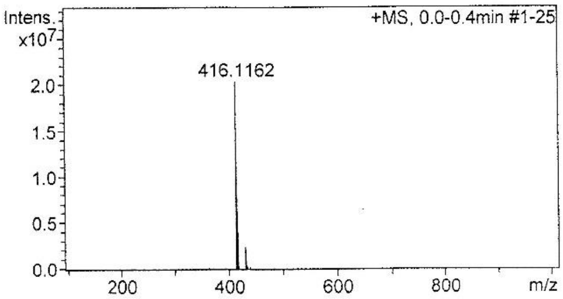

#	m/z	Res.	S/N	I	I %	FWHM
1	416.1162	27500	74658.7	20356332	100.0	0.0151
2	432.0899	44364	8952.9	2472999	12.1	0.0097

Figure 38. MS spectra of compound **4b**

Compound Spectrum List Report

Analysis Info

Analysis Name	D:\Data\KMCN.d	Acquisition Date	6/5/2017 1:21:20 PM
Method	low_mass.m	Operator	KM
Sample Name	TM Low concentration	Instrument	impact II 1825265.10082
Comment			

Acquisition Parameter

Source Type	ESI	Ion Polarity	Positive	Set Nebulizer	0.3 Bar
Focus	Active	Set Capillary	4000 V	Set Dry Heater	240 °C
Scan Begin	100 m/z	Set End Plate Offset	-500 V	Set Dry Gas	4.0 l/min
Scan End	1000 m/z	Set Charging Voltage	2000 V	Set Divert Valve	Source
		Set Corona	0 nA	Set APCI Heater	0 °C

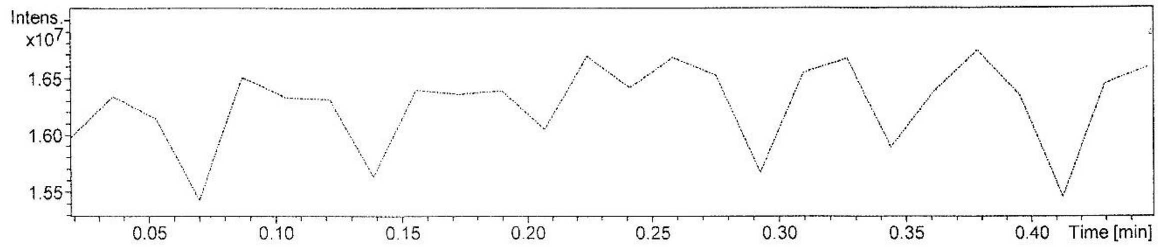

+MS, 0.0-0.4min #1-26

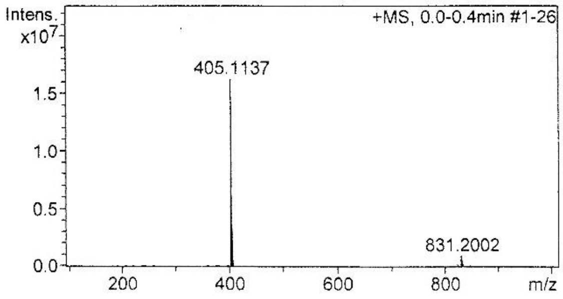

#	m/z	Res.	S/N	I	I %	FWHM
1	405.1137	44984	66402.4	16272570	100.0	0.0090
2	831.2002	45437	3748.5	1057117	15.5	0.0097

Figure 39. MS spectra of compound **5a**

Compound Spectrum List Report

Analysis Info

Analysis Name	D:\Data\KMMeCN_2.d	Acquisition Date	6/5/2017 11:31:27 AM	
Method	low_mass.m	Operator	KM	
Sample Name	TM Low concentration	Instrument	impact II	1825265.10082
Comment				

Acquisition Parameter

Source Type	ESI	Ion Polarity	Positive	Set Nebulizer	0.3 Bar
Focus	Active	Set Capillary	4000 V	Set Dry Heater	240 °C
Scan Begin	100 m/z	Set End Plate Offset	-500 V	Set Dry Gas	4.0 l/min
Scan End	1000 m/z	Set Charging Voltage	2000 V	Set Divert Valve	Source
		Set Corona	0 nA	Set APCI Heater	0 °C

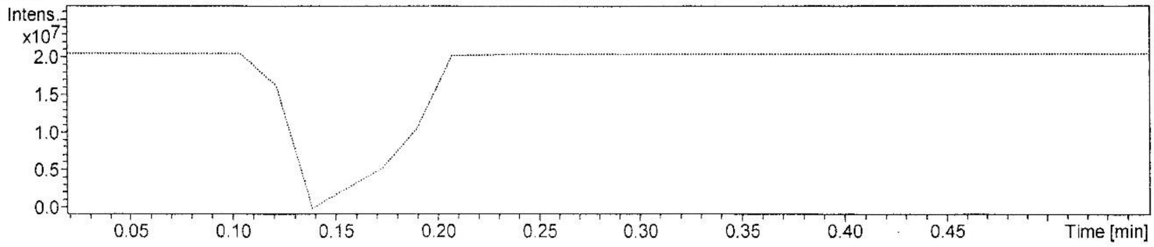

+MS, 0.0-0.5min #1-32

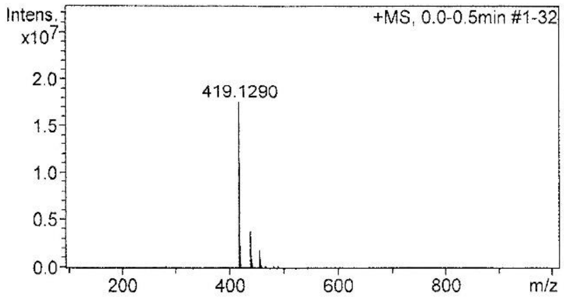

#	m/z	Res.	S/N	I	I %	FWHM
1	419.1290	35777	65498.4	17587406	100.0	0.0117
2	441.1108	46285	13858.6	3903586	22.2	0.0095
3	457.0646	44896	6677.4	1994532	11.3	0.0102

Figure 40. MS spectra of compound **5b**

Compound Spectrum List Report

Analysis Info

Analysis Name	D:\Data\KMFluor.d	Acquisition Date	6/5/2017 1:18:16 PM
Method	low_mass.m	Operator	KM
Sample Name	TM Low concentration	Instrument	impact II 1825265.10082
Comment			

Acquisition Parameter

Source Type	ESI	Ion Polarity	Positive	Set Nebulizer	0.3 Bar
Focus	Active	Set Capillary	4000 V	Set Dry Heater	240 °C
Scan Begin	100 m/z	Set End Plate Offset	-500 V	Set Dry Gas	4.0 l/min
Scan End	1000 m/z	Set Charging Voltage	2000 V	Set Divert Valve	Source
		Set Corona	0 nA	Set APCI Heater	0 °C

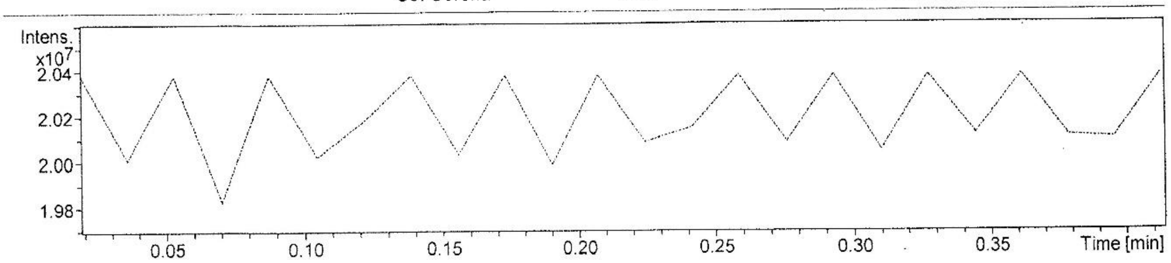

+MS, 0.0-0.4min #1-24

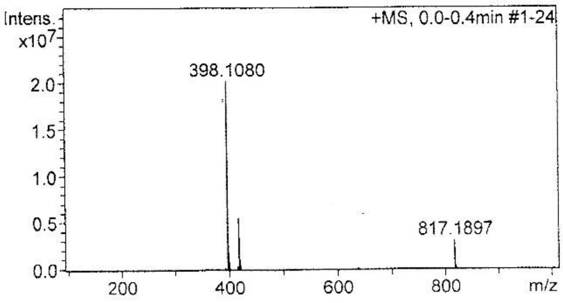

#	m/z	Res.	S/N	I	I %	FWHM
1	398.1080	18444	66336.3	20205136	100.0	0.0216
2	420.0896	45712	17474.5	5535816	27.4	0.0092
3	817.1897	54789	11893.4	3195751	15.8	0.0149

Figure 41. MS spectra of compound **6a**

Compound Spectrum List Report

Analysis Info

Acquisition Date 6/5/2017 11:35:15 AM

Analysis Name D:\Data\KMMeF.d
Method low_mass.m
Sample Name TM Low concentration
Comment

Operator KM
Instrument impact II 1825265.10082

Acquisition Parameter

Source Type	ESI	Ion Polarity	Positive	Set Nebulizer	0.3 Bar
Focus	Active	Set Capillary	4000 V	Set Dry Heater	240 °C
Scan Begin	100 m/z	Set End Plate Offset	-500 V	Set Dry Gas	4.0 l/min
Scan End	1000 m/z	Set Charging Voltage	2000 V	Set Divert Valve	Source
		Set Corona	0 nA	Set APCI Heater	0 °C

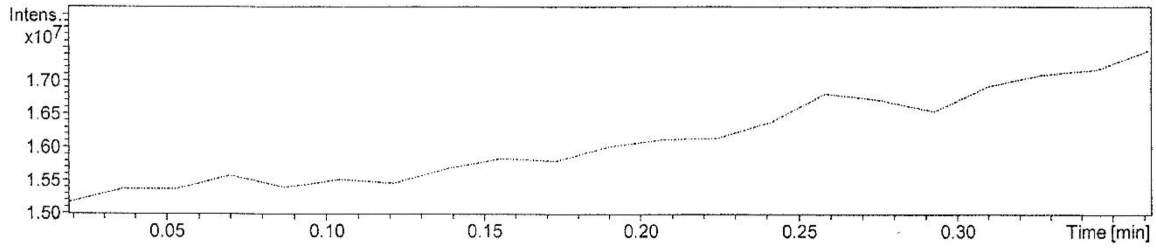

+MS, 0.0-0.4min #1-21

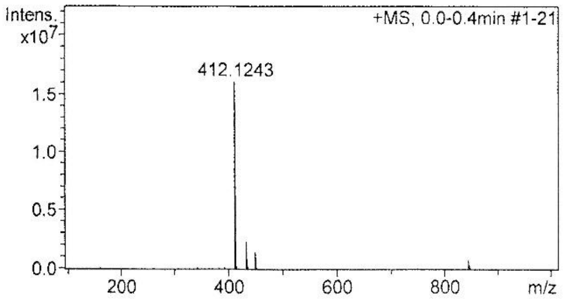

#	m/z	Res.	S/N	I	I %	FWHM
1	412.1243	45492	64544.0	16146119	100.0	0.0091
2	434.1059	44451	8622.6	2384991	14.8	0.0098
3	450.0798	42805	5033.8	1469029	9.1	0.0105

Figure 42. MS spectra of compound **6b**

Compound Spectrum List Report

Analysis Info

Analysis Name	D:\Data\KMNO2.d	Acquisition Date	6/5/2017 1:38:54 PM
Method	low_mass.m	Operator	KM
Sample Name	TM Low concentration	Instrument	impact II 1825265.10082
Comment			

Acquisition Parameter

Source Type	ESI	Ion Polarity	Positive	Set Nebulizer	0.3 Bar
Focus	Active	Set Capillary	4000 V	Set Dry Heater	240 °C
Scan Begin	100 m/z	Set End Plate Offset	-500 V	Set Dry Gas	4.0 l/min
Scan End	1000 m/z	Set Charging Voltage	2000 V	Set Divert Valve	Source
		Set Corona	0 nA	Set APCI Heater	0 °C

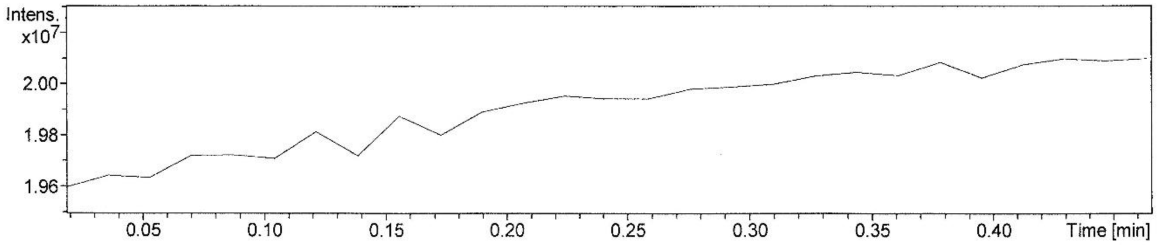

+MS, 0.0-0.5min #1-27

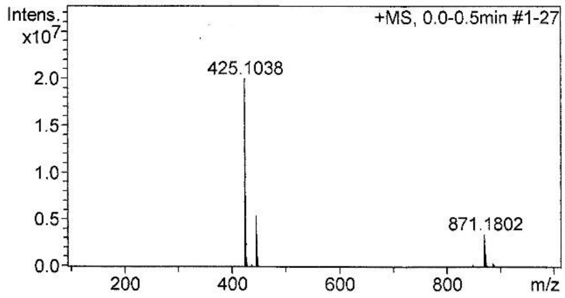

#	m/z	Res.	S/N	I	I %	FWHM
1	425.1038	36271	91580.0	19904348	100.0	0.0117
2	447.0855	46705	22943.2	5479667	27.5	0.0096
3	463.0591	42196	6064.8	3551317	17.8	0.0155

Figure 43. MS spectra of compound **7a**

Compound Spectrum List Report

Analysis Info

Analysis Name	D:\Data\KMMeNO2.d	Acquisition Date	6/5/2017 11:38:39 AM
Method	low_mass.m	Operator	KM
Sample Name	TM Low concentration	Instrument	impact II 1825265.10082
Comment			

Acquisition Parameter

Source Type	ESI	Ion Polarity	Positive	Set Nebulizer	0.3 Bar
Focus	Active	Set Capillary	4000 V	Set Dry Heater	240 °C
Scan Begin	100 m/z	Set End Plate Offset	-500 V	Set Dry Gas	4.0 l/min
Scan End	1000 m/z	Set Charging Voltage	2000 V	Set Divert Valve	Source
		Set Corona	0 nA	Set APCI Heater	0 °C

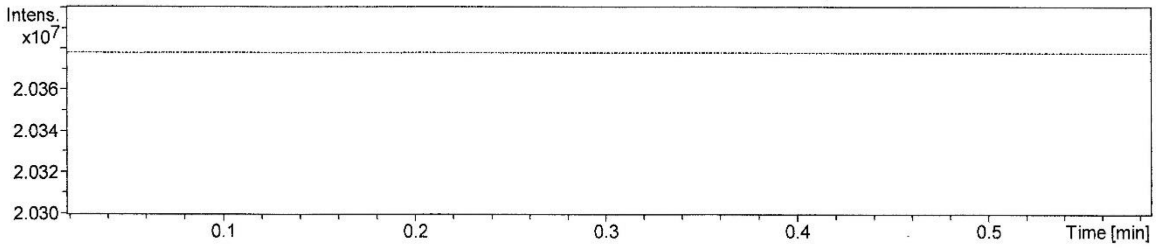

+MS, 0.0-0.6min #1-34

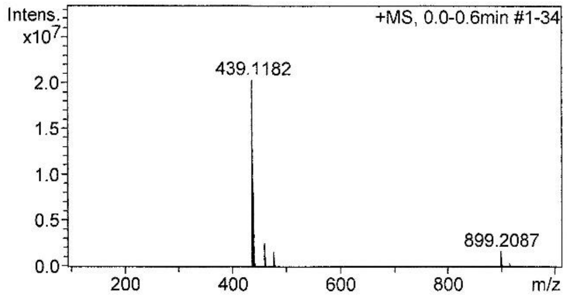

#	m/z	Res.	S/N	I	I %	FWHM
1	439.1182	20251	58925.2	20377588	100.0	0.0217
2	461.0997	45729	7339.8	2628508	12.9	0.0101
3	477.0735	43955	4379.6	1598306	7.8	0.0109
4	899.2087	51205	8262.2	1765546	8.7	0.0176

Figure 44. MS spectra of compound **7b**

Compound Spectrum List Report

Analysis Info

Acquisition Date 6/5/2017 1:27:00 PM

Analysis Name D:\Data\KMMTM.d

Method low_mass.m

Sample Name TM Low concentration

Comment

Operator KM

Instrument impact II 1825265.10082

Acquisition Parameter

Source Type	ESI	Ion Polarity	Positive	Set Nebulizer	0.3 Bar
Focus	Active	Set Capillary	4000 V	Set Dry Heater	240 °C
Scan Begin	100 m/z	Set End Plate Offset	-500 V	Set Dry Gas	4.0 l/min
Scan End	1000 m/z	Set Charging Voltage	2000 V	Set Divert Valve	Source
		Set Corona	0 nA	Set APCI Heater	0 °C

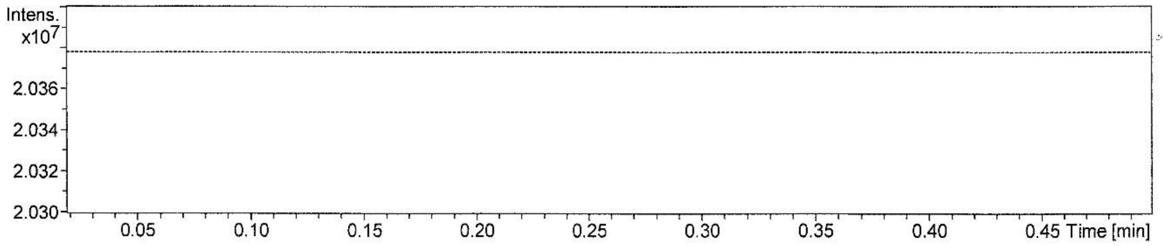

+MS, 0.0-0.5min #1-29

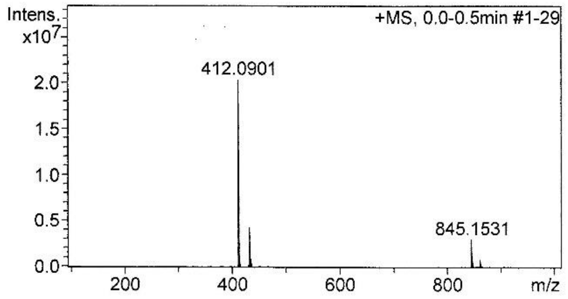

#	m/z	Res.	S/N	I	I %	FWHM
1	412.0901	17841	72612.0	20377596	100.0	0.0231
2	434.0715	45782	14979.6	4285151	21.0	0.0095
3	845.1531	54598	13920.8	3105717	15.2	0.0155

Figure 45. MS spectra of compound **8a**

Compound Spectrum List Report

Analysis Info

Acquisition Date 6/5/2017 11:43:20 AM

Analysis Name D:\Data\KMMeMTM.d
Method low_mass.m
Sample Name TM Low concentration
Comment

Operator KM
Instrument impact II 1825265.10082

Acquisition Parameter

Source Type	ESI	Ion Polarity	Positive	Set Nebulizer	0.3 Bar
Focus	Active	Set Capillary	4000 V	Set Dry Heater	240 °C
Scan Begin	100 m/z	Set End Plate Offset	-500 V	Set Dry Gas	4.0 l/min
Scan End	1000 m/z	Set Charging Voltage	2000 V	Set Divert Valve	Source
		Set Corona	0 nA	Set APCI Heater	0 °C

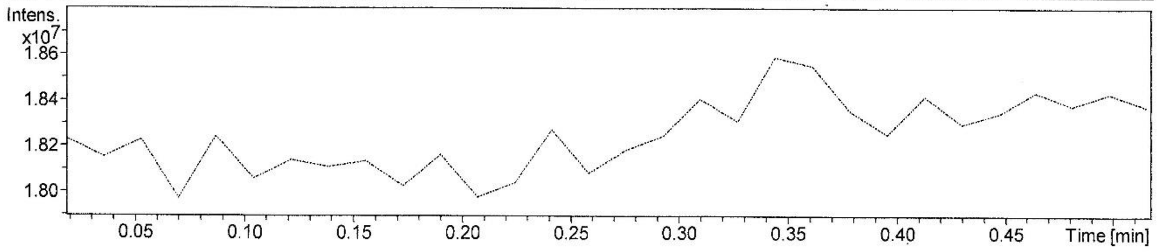

+MS, 0.0-0.5min #1-30

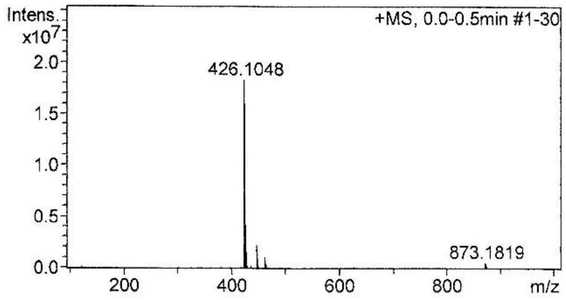

#	m/z	Res.	S/N	I	I %	FWHM
1	426.1048	43867	58820.5	18242236	100.0	0.0097
2	448.0863	43755	6764.9	2331985	12.8	0.0102
3	464.0601	41318	3202.0	1164486	6.4	0.0112
4	873.1819	42257	2731.7	642415	3.5	0.0207

Figure 46. MS spectra of compound **8b**

Compound Spectrum List Report

**Analysis Info**			
Analysis Name	D:\Data\KMMe7QCl.d	Acquisition Date	6/5/2017 11:47:57 AM
Method	low_mass.m	Operator	KM
Sample Name	TM Low concentration	Instrument	impact II 1825265.10082
Comment			

Acquisition Parameter

Source Type	ESI	Ion Polarity	Positive	Set Nebulizer	0.3 Bar
Focus	Active	Set Capillary	4000 V	Set Dry Heater	240 °C
Scan Begin	100 m/z	Set End Plate Offset	-500 V	Set Dry Gas	4.0 l/min
Scan End	1000 m/z	Set Charging Voltage	2000 V	Set Divert Valve	Source
		Set Corona	0 nA	Set APCI Heater	0 °C

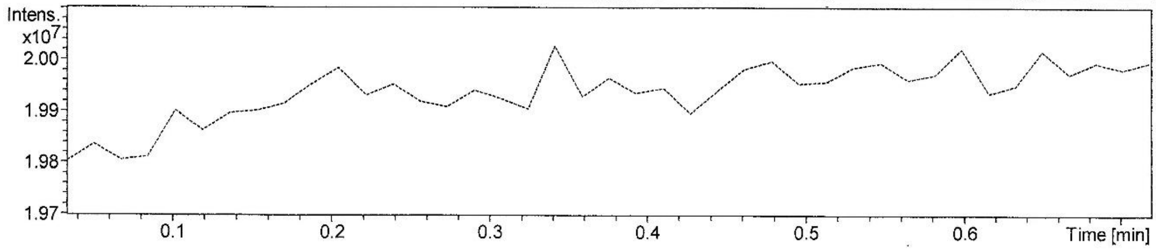

+MS, 0.0-0.7min #1-41

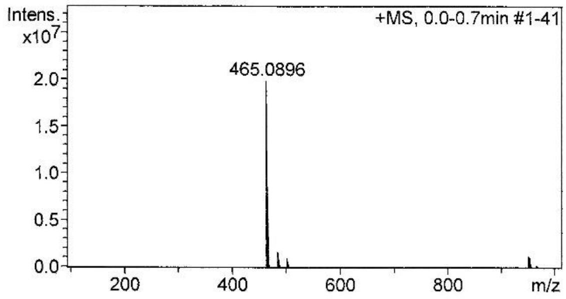

#	m/z	Res.	S/N	I	I %	FWHM
1	465.0896	35286	49119.5	19926644	100.0	0.0132
2	487.0710	42681	4193.6	1770303	8.9	0.0114
3	503.0448	41308	2277.9	987557	5.0	0.0122
4	951.1505	47625	3649.5	1307621	6.6	0.0200

Figure 47. MS spectra of compound **9b**

Compound Spectrum List Report

Analysis Info

Analysis Name	D:\Data\KMMe8QBr.d
Method	low_mass.m
Sample Name	TM Low concentration
Comment	

Acquisition Date 6/5/2017 11:51:55 AM

Operator KM
Instrument impact II 1825265.10082

Acquisition Parameter

Source Type	ESI	Ion Polarity	Positive	Set Nebulizer	0.3 Bar
Focus	Active	Set Capillary	4000 V	Set Dry Heater	240 °C
Scan Begin	100 m/z	Set End Plate Offset	-500 V	Set Dry Gas	4.0 l/min
Scan End	1000 m/z	Set Charging Voltage	2000 V	Set Divert Valve	Source
		Set Corona	0 nA	Set APCI Heater	0 °C

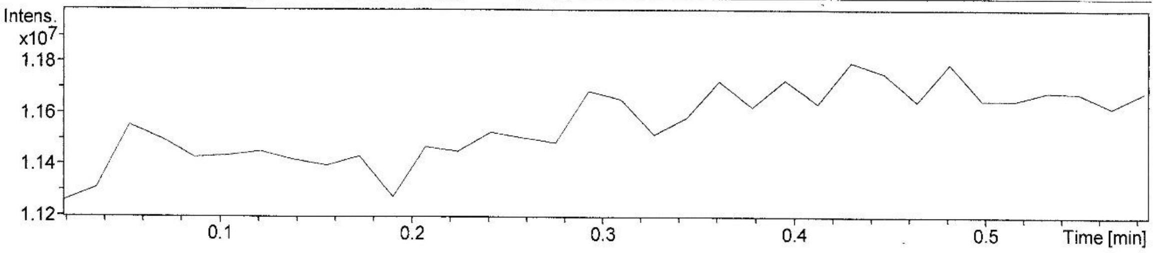

+MS, 0.0-0.6min #1-34

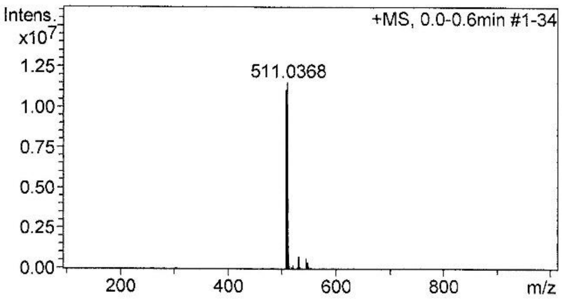

#	m/z	Res.	S/N	I	I %	FWHM
1	509.0388	50360	38148.9	11093930	96.0	0.0101
2	511.0368	50670	39607.8	11559695	100.0	0.0101
3	531.0200	38349	2474.8	741308	6.4	0.0138
4	533.0182	38791	2601.1	779352	6.7	0.0137
5	546.9939	36436	1279.0	390372	3.4	0.0150
6	548.9920	35172	1361.5	415778	3.6	0.0156

Figure 48. MS spectra of compound **10a**

Compound Spectrum List Report

Analysis Info

Analysis Name	D:\Data\KMMe8QBr.d	Acquisition Date	6/5/2017 11:51:55 AM
Method	low_mass.m	Operator	KM
Sample Name	TM Low concentration	Instrument	impact II 1825265.10082
Comment			

Acquisition Parameter

Source Type	ESI	Ion Polarity	Positive	Set Nebulizer	0.3 Bar
Focus	Active	Set Capillary	4000 V	Set Dry Heater	240 °C
Scan Begin	100 m/z	Set End Plate Offset	-500 V	Set Dry Gas	4.0 l/min
Scan End	1000 m/z	Set Charging Voltage	2000 V	Set Divert Valve	Source
		Set Corona	0 nA	Set APCI Heater	0 °C

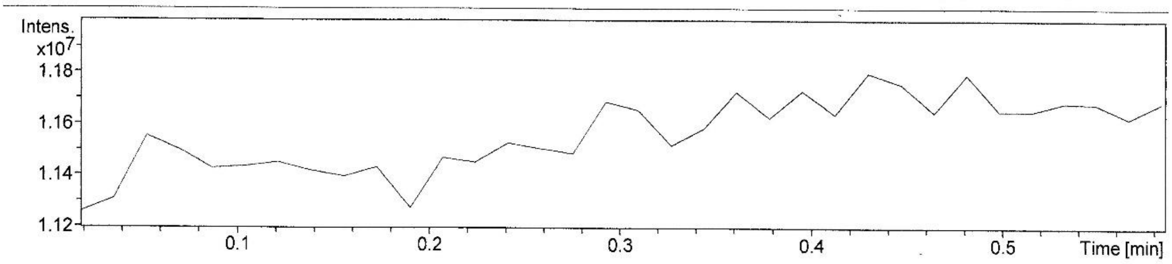

+MS, 0.0-0.6min #1-34

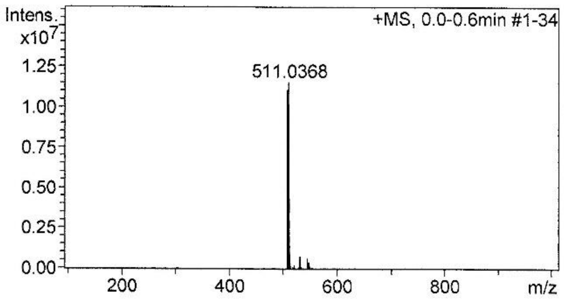

#	m/z	Res.	S/N	I	I %	FWHM
1	509.0388	50360	38148.9	11093930	96.0	0.0101
2	511.0368	50670	39607.8	11559695	100.0	0.0101
3	531.0200	38349	2474.8	741308	6.4	0.0138
4	533.0182	38791	2601.1	779352	6.7	0.0137
5	546.9939	36436	1279.0	390372	3.4	0.0150
6	548.9920	35172	1361.5	415778	3.6	0.0156

Figure 49. MS spectra of compound **10b**

Compound Spectrum List Report

Analysis Info

Analysis Name	D:\Data\KMAZT.d	Acquisition Date	6/5/2017 1:30:02 PM
Method	low_mass.m	Operator	KM
Sample Name	TM Low concentration	Instrument	impact II 1825265.10082
Comment			

Acquisition Parameter

Source Type	ESI	Ion Polarity	Positive	Set Nebulizer	0.3 Bar
Focus	Active	Set Capillary	4000 V	Set Dry Heater	240 °C
Scan Begin	100 m/z	Set End Plate Offset	-500 V	Set Dry Gas	4.0 l/min
Scan End	1000 m/z	Set Charging Voltage	2000 V	Set Divert Valve	Source
		Set Corona	0 nA	Set APCI Heater	0 °C

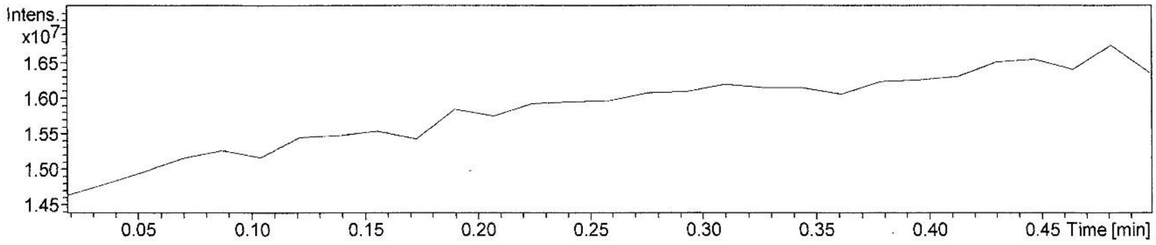

+MS, 0.0-0.5min #1-29

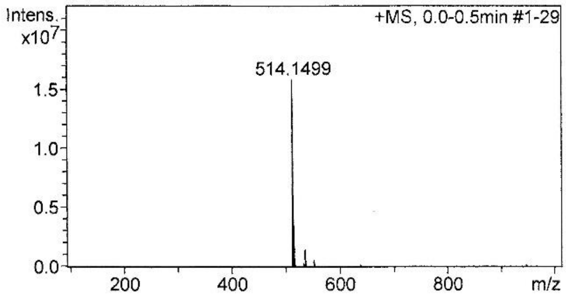

#	m/z	Res.	S/N	I	I %	FWHM
1	514.1499	50203	56898.7	15858082	100.0	0.0102
2	536.1312	42976	5087.3	1479755	9.3	0.0125
3	552.1049	37520	1906.1	568949	3.6	0.0147

Figure 50. MS spectra of compound **11a**

Compound Spectrum List Report

Analysis Info

Analysis Name	D:\Data\KMMeAZT.d	Acquisition Date	6/5/2017 11:59:17 AM
Method	low_mass.m	Operator	KM
Sample Name	TM Low concentration	Instrument	impact II 1825265.10082
Comment			

Acquisition Parameter

Source Type	ESI	Ion Polarity	Positive	Set Nebulizer	0.3 Bar
Focus	Active	Set Capillary	4000 V	Set Dry Heater	240 °C
Scan Begin	100 m/z	Set End Plate Offset	-500 V	Set Dry Gas	4.0 l/min
Scan End	1000 m/z	Set Charging Voltage	2000 V	Set Divert Valve	Source
		Set Corona	0 nA	Set APCI Heater	0 °C

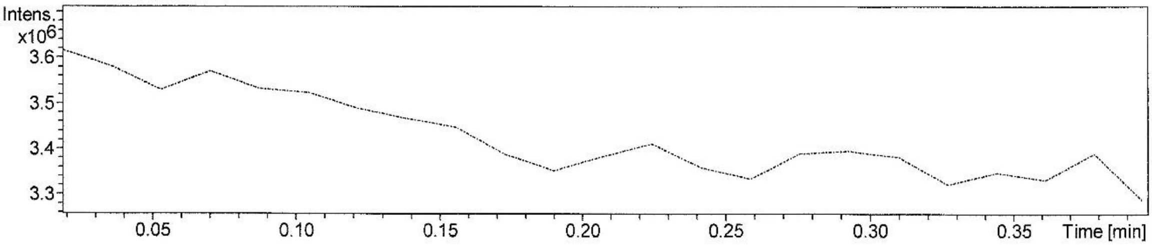

+MS, 0.0-0.4min #1-23

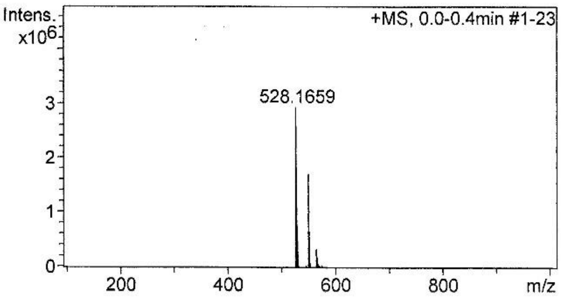

#	m/z	Res.	S/N	I	I %	FWHM
1	528.1659	48254	10640.7	2936504	100.0	0.0109
2	550.1476	43867	5921.1	1714562	58.4	0.0125
3	566.1214	36237	1119.3	334057	11.4	0.0156

Figure 51. MS spectra of compound **11b**

Compound Spectrum List Report

Analysis Info

Analysis Name	D:\Data\KMMeButyl.d	Acquisition Date	6/5/2017 12:17:35 PM
Method	low_mass.m	Operator	KM
Sample Name	TM Low concentration	Instrument	impact II 1825265.10082
Comment			

Acquisition Parameter

Source Type	ESI	Ion Polarity	Positive	Set Nebulizer	0.3 Bar
Focus	Active	Set Capillary	4000 V	Set Dry Heater	240 °C
Scan Begin	100 m/z	Set End Plate Offset	-500 V	Set Dry Gas	4.0 l/min
Scan End	1000 m/z	Set Charging Voltage	2000 V	Set Divert Valve	Source
		Set Corona	0 nA	Set APCI Heater	0 °C

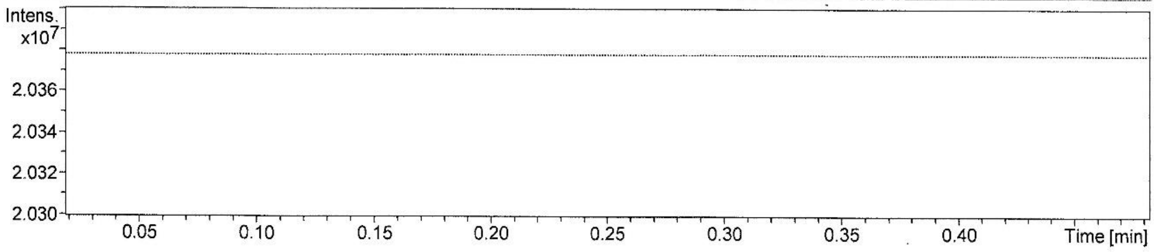

+MS, 0.0-0.5min #1-28

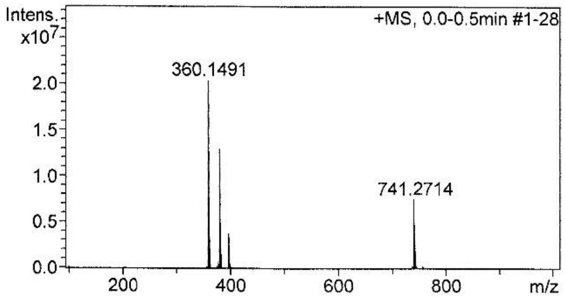

#	m/z	Res.	S/N	I	I %	FWHM
1	360.1491	13805	26118.7	20377596	100.0	0.0261
2	382.1300	43913	15999.9	12913806	63.4	0.0087
3	398.1039	44078	4728.7	3769924	18.5	0.0090
4	741.2714	57999	16284.5	7588718	37.2	0.0128

Figure 52. MS spectra of compound **12b**

Compound Spectrum List Report

Analysis Info

Analysis Name	D:\Data\KMMeEster.d	Acquisition Date	6/5/2017 12:13:48 PM
Method	low_mass.m	Operator	KM
Sample Name	TM Low concentration	Instrument	impact II 1825265.10082
Comment			

Acquisition Parameter

Source Type	ESI	Ion Polarity	Positive	Set Nebulizer	0.3 Bar
Focus	Active	Set Capillary	4000 V	Set Dry Heater	240 °C
Scan Begin	100 m/z	Set End Plate Offset	-500 V	Set Dry Gas	4.0 l/min
Scan End	1000 m/z	Set Charging Voltage	2000 V	Set Divert Valve	Source
		Set Corona	0 nA	Set APCI Heater	0 °C

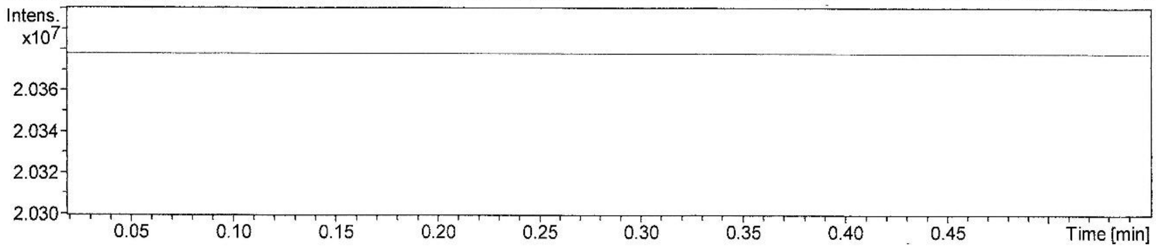

+MS, 0.0-0.5min #1-32

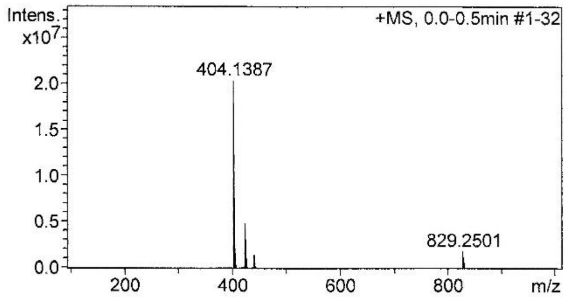

#	m/z	Res.	S/N	I	I %	FWHM
1	404.1387	16873	35993.8	20377596	100.0	0.0240
2	426.1200	45545	8292.2	4904802	24.1	0.0094
3	442.0937	42409	2592.9	1558017	7.6	0.0104
4	829.2501	51700	6399.4	1913042	9.4	0.0160

Figure 53. MS spectra of compound **13b**

Compound Spectrum List Report

Analysis Info

Acquisition Date 6/5/2017 1:50:29 PM

Analysis Name	D:\Data\KMBetulina.d			
Method	low_mass.m	Operator	KM	
Sample Name	TM Low concentration	Instrument	impact II	1825265.10082
Comment				

Acquisition Parameter

Source Type	ESI	Ion Polarity	Positive	Set Nebulizer	0.3 Bar
Focus	Active	Set Capillary	4000 V	Set Dry Heater	240 °C
Scan Begin	100 m/z	Set End Plate Offset	-500 V	Set Dry Gas	4.0 l/min
Scan End	1000 m/z	Set Charging Voltage	2000 V	Set Divert Valve	Source
		Set Corona	0 nA	Set APCI Heater	0 °C

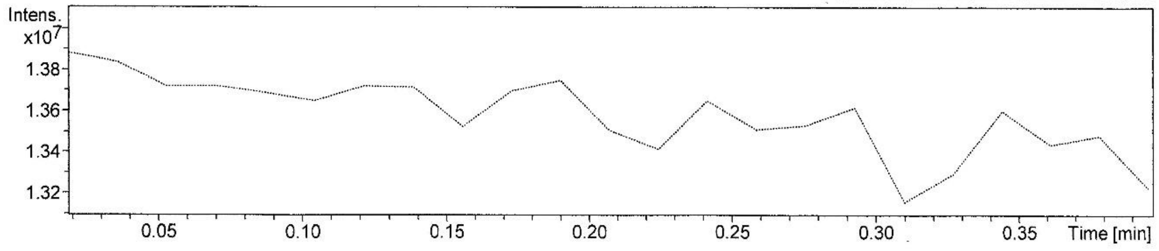

+MS, 0.0-0.4min #1-23

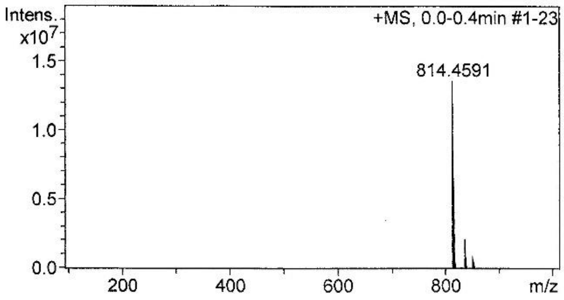

#	m/z	Res.	S/N	I	I %	FWHM
1	814.4591	61791	63445.3	13576690	100.0	0.0132
2	836.4400	52010	10165.5	2179482	16.1	0.0161
3	852.4137	40011	3226.9	689647	5.1	0.0213

Figure 54. MS spectra of compound **14a**

Table S1. Structures of compounds used in the in silico study

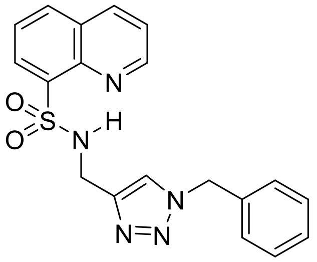	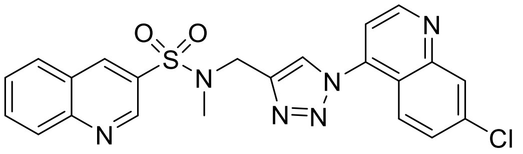
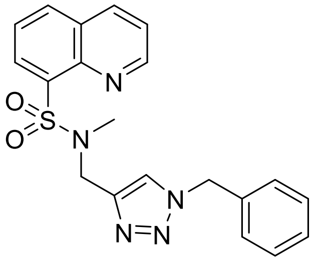	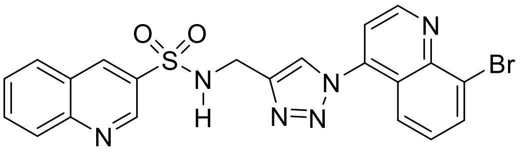
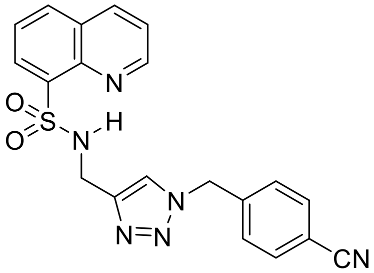	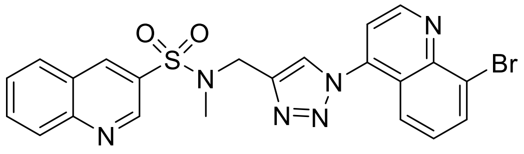
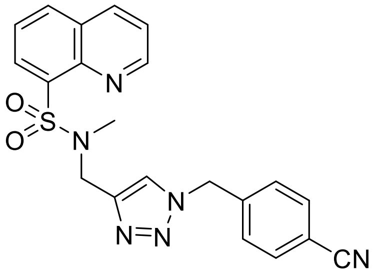	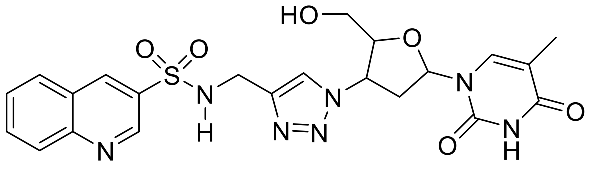
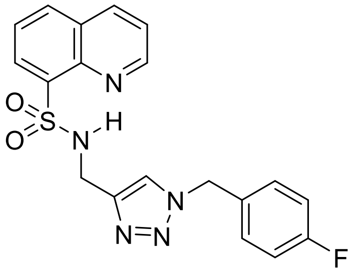	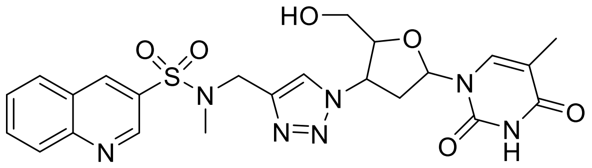
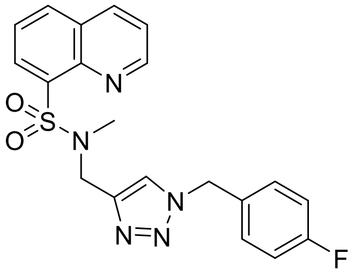	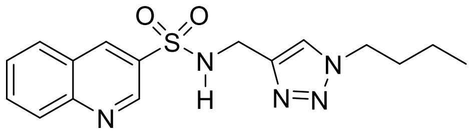
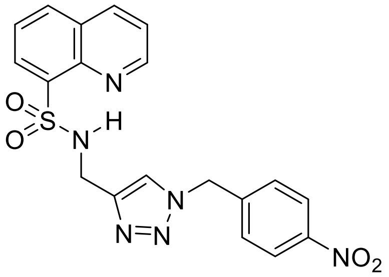	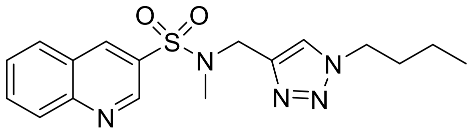

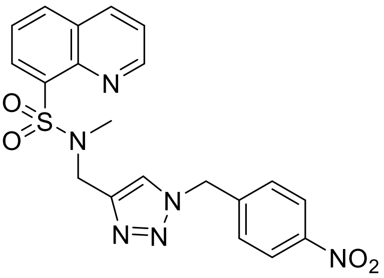	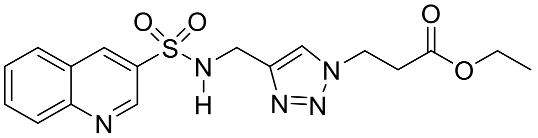
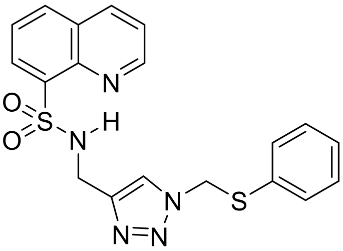	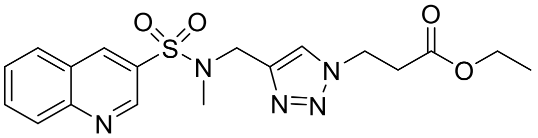
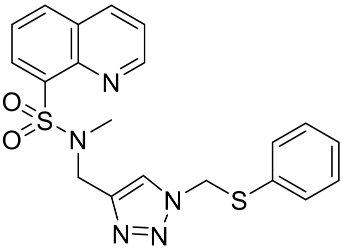	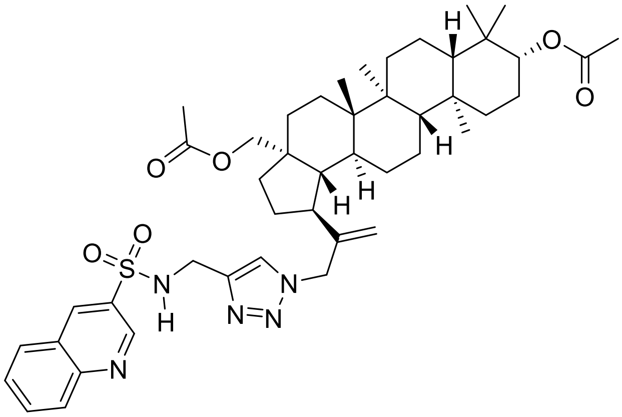
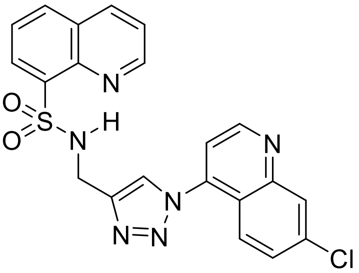	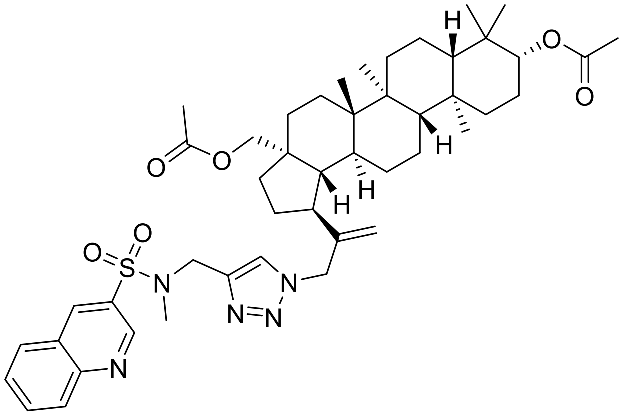
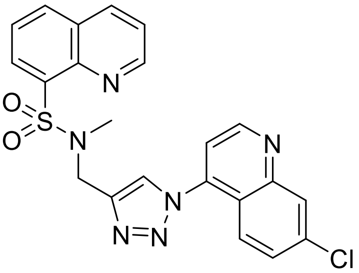	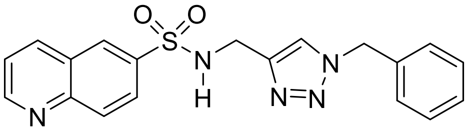
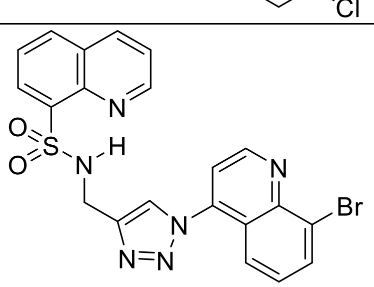	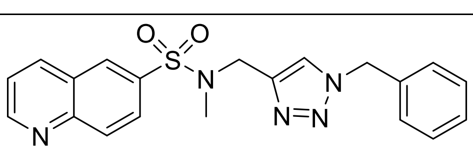

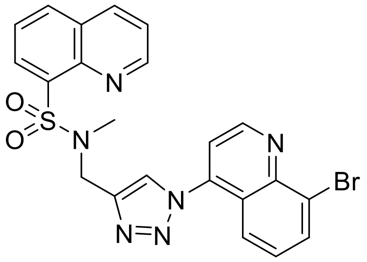	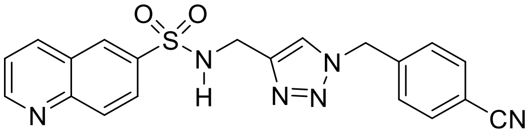
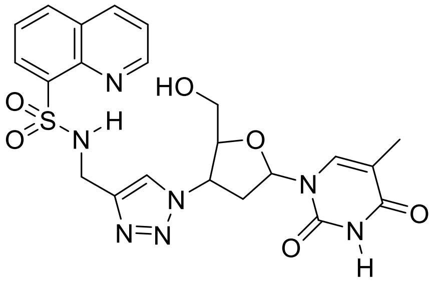	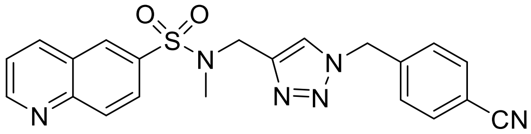
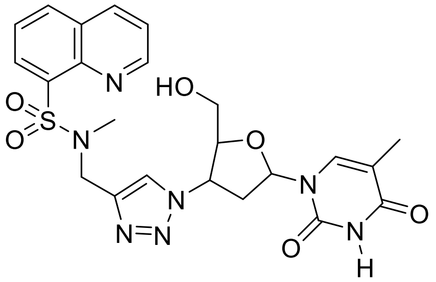	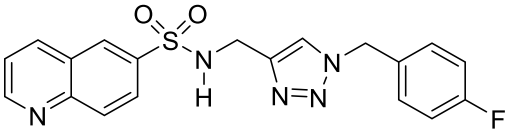
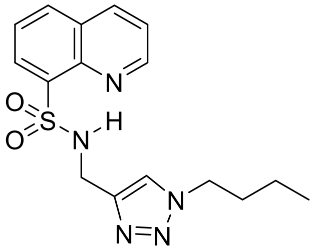	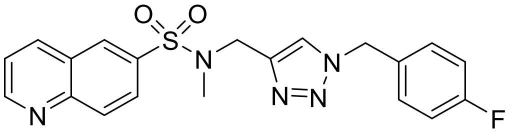
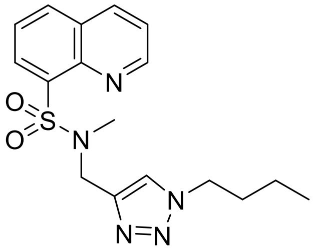	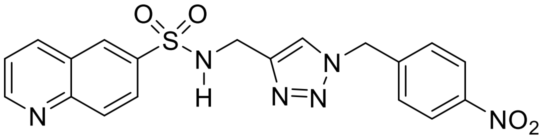
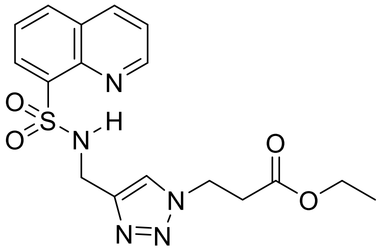	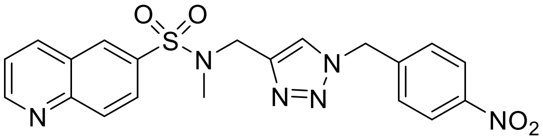
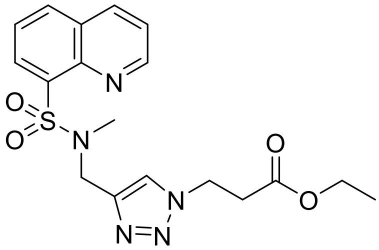	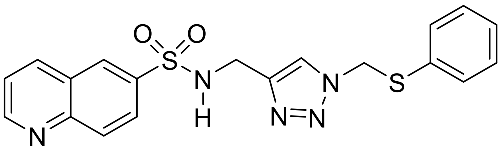

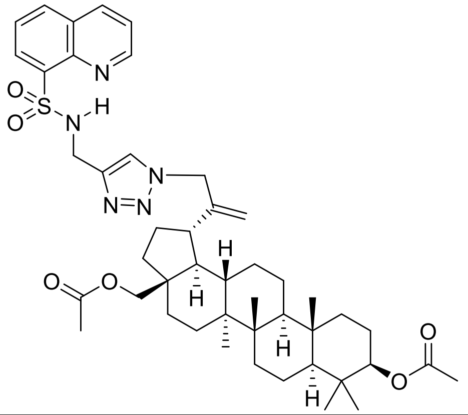	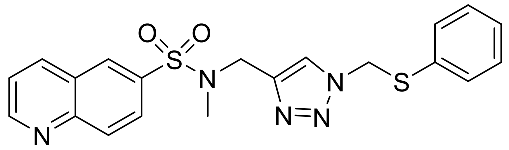
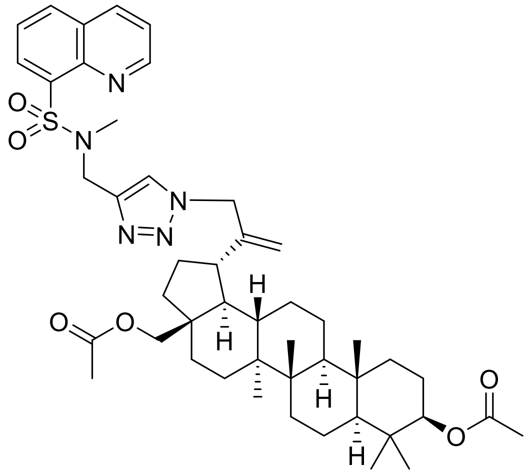	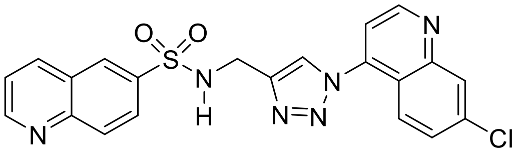
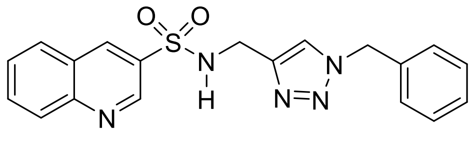	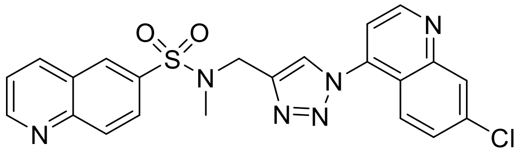
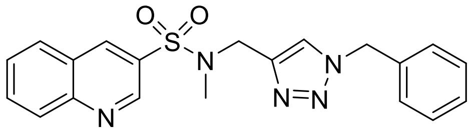	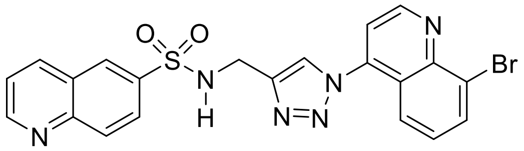
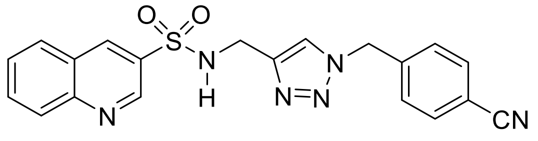	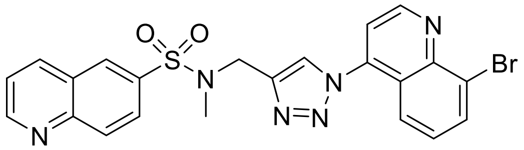
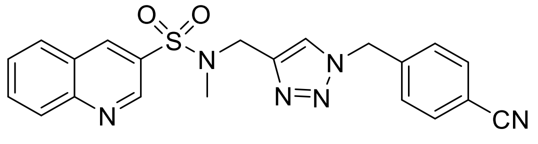	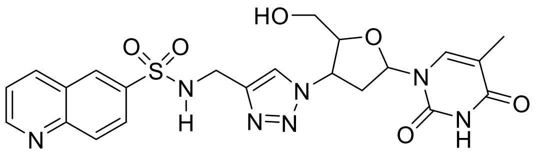
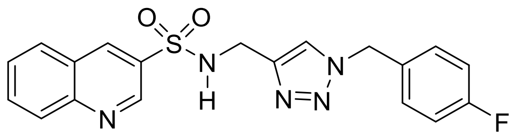	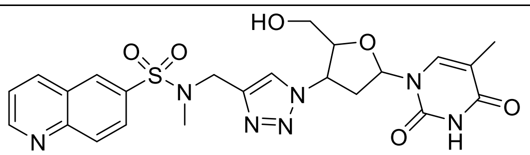
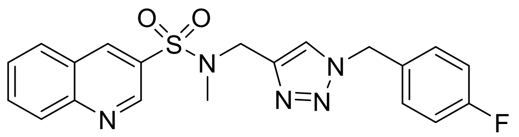	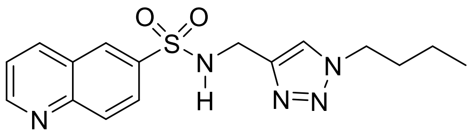
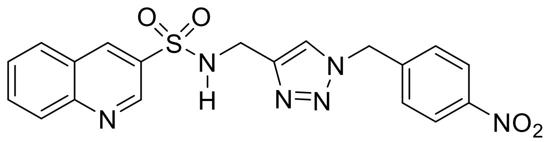	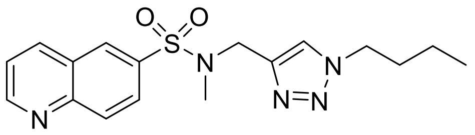

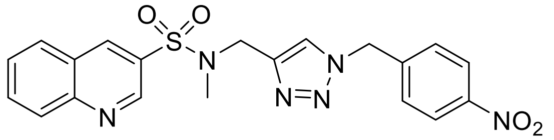	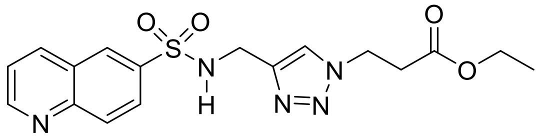
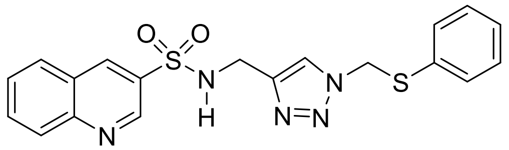	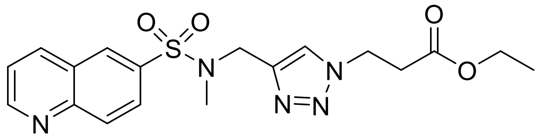
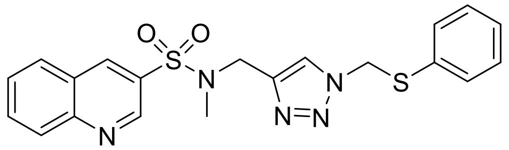	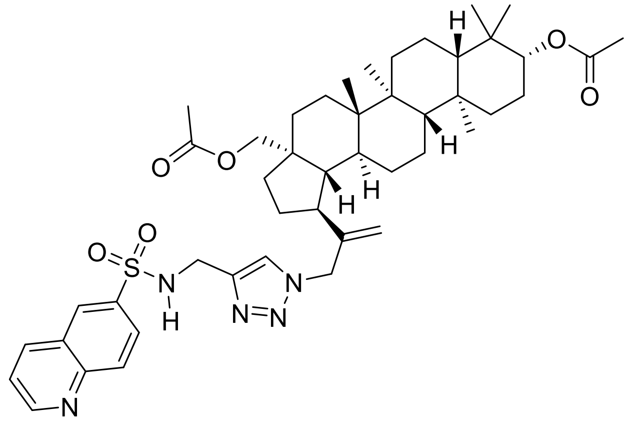
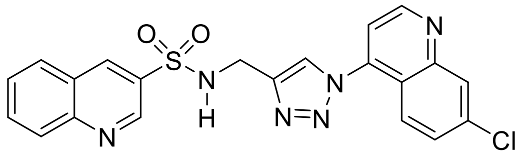	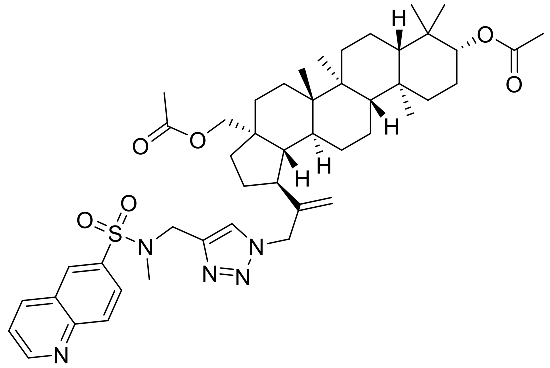
